# Genome and transcriptome analyses reveal molecular mechanisms underlying the interaction between *Plasmopara viticola* and grapevine

**DOI:** 10.3389/fpls.2026.1765002

**Published:** 2026-03-10

**Authors:** R. Karan, M. K. Prasannakumar, Kiran B. M., J. Harish, B. Roopashree, Gopal Venkateshbabu, Swathi S. Patil, S. Shreedevasena, H. B. Mahesh, Pramesh Devanna, C. Manjunatha, Aditya Kukreti, Aditya Narayan Sarangi, Raju Soolanayakanahally, Sateesh Kagle

**Affiliations:** 1 PathoGenOmics Lab, Department of Plant Pathology, University of Agricultural Sciences, Bangalore, Karnataka, India; 2 ICAR-Directorate of Medicinal and Aromatic Plants Research, Anand, India; 3 Department of Genetics and Plant Breeding, University of Agricultural Sciences, Bangalore, Karnataka, India; 4 ICAR-Directorate of Mushroom Research, Solan, India; 5 ICAR-National Bureau of Agricultural Insect Resources, Bengaluru, India; 6 BaseSolve Informatics Pvt. Ltd., Ahmedabad, Gujarat, India; 7 Saskatoon Research and Development Centre, Agriculture and Agri-Food Canada, Saskatoon, SK, Canada; 8 Aquatic and Crop Resource Development, National Research Council Canada, Saskatoon, SK, Canada

**Keywords:** downy mildew, effectors, genome, grapes, grapevine, *Plasmopara viticola*, transcriptome

## Abstract

*Plasmopara viticola*, an obligate biotrophic oomycete, is the causal agent of downy mildew in grapevine (*Vitis vinifera*) and a major constraint to viticulture worldwide. Here, we report the first high-quality whole-genome assembly of an Indian *P. viticola* isolate (PV01), generated using a hybrid sequencing approach combining Illumina and Oxford Nanopore platforms. The assembled genome spans 84.09 Mb across 182 contigs, with an N50 of ~971 kb and 97% BUSCO completeness, and encodes 12,404 predicted protein-coding genes, diverse transposable elements, and lineage-specific expansions. Functional annotation revealed a rich repertoire of effectors, including RXLR, CRN, and apoplastic effectors, as well as putative virulence-associated and secretory proteins likely involved in host manipulation and immune suppression. Comparative ortholog analysis across *P. viticola* isolates and representative oomycetes identified a conserved core genome alongside 164 PV01-specific orthogroups, reflecting isolate-level diversification. Dual RNA-seq analysis of infected grapevine leaves revealed strong suppression of chloroplast- and photosynthesis-associated pathways in the host, coupled with induction of defense-related genes, including PR proteins, WRKY transcription factors, calcium signaling components, and JA/ET-mediated pathways. Concurrently, *P. viticola* displayed infection-stage–specific expression of effectors, apoplastic proteases, vesicle trafficking components, and genes associated with autophagy suppression and redox homeostasis. Together, these integrated genomic and transcriptomic analyses provide insights into the molecular mechanisms underlying *P. viticola* pathogenicity and grapevine immune modulation.

## Introduction

The grapevine (*Vitis vinifera* L.) was among the earliest domesticated fruit crops and remains one of the most economically significant horticultural crop worldwide. Grapevine cultivation is central to the wine industry and contributes substantially to the agricultural economies of many nations. However, viticulture faces persistent threats from devastating pathogens that reduce yield and quality (Meiri and Bar-Oz, 2024).

Among these, downy mildew, caused by the obligate biotrophic oomycete *Plasmopara viticola* (Berk. & Curt.) Berl. & de Toni, is one of the most destructive diseases in grape-growing regions globally. The pathogen infects members of the Vitaceae family, particularly the cultivated species *V. vinifera*, and attacks all aerial green tissues including leaves, shoots, inflorescences, and berries (Buonassisi et al., 2018) (Vezzulli et al., 2018). Typical symptoms include chlorotic “oil spots” on the adaxial leaf surface and dense white sporulation on the abaxial side under favorable conditions of high humidity (93%) and mild temperatures (18-20 °C), ultimately leading to defoliation, poor fruit set, and severe crop loss (Yıldırım-Yalçın et al., 2019) (Nogueira Júnior et al., 2020).

*P. viticola* originated in North America and was introduced to Europe in the late 19th century, where it rapidly spread and devastated viticulture (Koledenkova et al., 2022). Population studies have since revealed marked genetic diversity and host specialization across continents, with distinct clusters identified in North America, Europe, and, more recently in Asia (Rumbou and Gessler, 2004, 2006; Ghule et al., 2018). The pathogen is heterothallic and undergoes frequent sexual recombination, generating diverse, panmictic populations that accelerate adaptive evolution (Gobbin et al., 2006). Consequently, fungicide resistance has emerged as a major management challenge, with isolates resistant to multiple site-specific fungicides, including kresoxim-methyl, dimethomorph, cymoxanil, and metalaxyl-M, reported from vineyards worldwide (Ghule et al., 2018). This evolutionary plasticity highlights the urgent need for durable, genetic resistance in grapevine.

While most *V. vinifera* cultivars remain highly susceptible to *P. viticola*, resistance has been identified in wild *Vitis* spp. from the Caucasus and North America (Toffolatti et al., 2018). Host resistance responses involve recognition of pathogen-associated molecular patterns (PAMPs) or pathogen effectors by host receptors, triggering signaling cascades and defense responses analogous to the innate immune system (Dong et al., 2011). These include accumulation of stilbene phytoalexins, lignification, callose deposition, oxidative bursts, and hypersensitive cell death to restrict pathogen spread (Li et al., 2015b) (Yin et al., 2017b) (Qu et al., 2021). Understanding the molecular interplay between grapevine defense mechanisms and pathogen virulence strategies is therefore central to breeding durable resistance.

Advances in genomics and transcriptomics have transformed our understanding of oomycete pathogens. Genomic resources are essential for understanding the pathogenic mechanisms, host adaptation and rapid evolutionary dynamics of *P. viticola*, an obligate biotrophic oomycete pathogen (Gouveia et al., 2024). Over the last decade, several genome assemblies of *P. viticola* have been published, reflecting steady improvements in sequencing technologies and genome assembly approaches.

The draft genome of *P. viticola* was generated using Illumina sequencing for the isolate INRA-PV221 with an estimated genome size of 74.7 Mb (Dussert et al., 2016). This assembly served as a foundational genomic resource and enabled early investigations into effector repertoires and population genomics of the pathogen. Subsequently, Yin et al. (2017a) reported a hybrid Illumina–PacBio genome assembly for isolate JL-7–2 with an expanded genome size of approximately 101.3 Mb. The inclusion of long-read sequencing substantially improved the assembly of repetitive regions and facilitated a more comprehensive characterization of the secretome, particularly RXLR and CRN effector families.

More recently, a high-quality genome assembly based exclusively on PacBio long-read sequencing was produced for *P. viticola*, with a genome size of 92.9 Mb and markedly enhanced contiguity (Dussert et al., 2019). This study revealed a distinct “two-speed genome” architecture, in which rapidly evolving effector genes are preferentially located in repeat-rich, gene-sparse regions, while conserved housekeeping genes are concentrated in gene-dense regions. Such genome organization is now widely recognized as a key driver of host adaptation, virulence evolution, and genomic plasticity in oomycete plant pathogens (Wacker et al., 2023).

Genome assemblies of *P. viticola* have revealed a compact, repeat-rich genome encoding a vast repertoire of secreted effector proteins, including RxLR effectors and CRN (Crinkler) proteins (Yin et al., 2017a) (Dussert et al., 2019), which are thought to modulate host immunity and facilitate biotrophy. Comparative analyses highlight convergent effector evolution across downy mildew pathogens, pointing to their critical role in shaping host-pathogen interactions and host specialization (Gouveia et al., 2024). However, detailed insights into the effector repertoire of *P. viticola* and their transcriptional regulation during infection remain limited (Chen et al., 2020).

In this study, we present an integrated analysis of the *P. viticola* genome and transcriptome, with emphasis on the early transcriptional dynamics of infection in *V vinifera* plants. By identifying candidate effector genes and dissecting their potential roles in virulence, we provide key insights into the molecular mechanisms underlying downy mildew pathogenicity. These findings not only advance our understanding of oomycete biology but also offer valuable resources for the development of novel, durable strategies for disease control and resistance breeding in grapevine.

## Materials and methods

### Sample collection and morphological characterization

Grapes leaves showing the characteristic symptoms of downy mildew were collected from Chikkaballapur and Bengaluru rural districts of Karnataka, India. All samples were placed in a humid chamber using 200 × 20 mm Petri dishes containing moist blotter paper to encourage vigorous sporulation. After 24 hours of incubation, leaves showing sporulation were selected, thin sectioned and observed under a light microscope. Subsequently, samples were subjected to scanning electron microscopy (SEM) to observe detailed spore structures.

### DNA isolation and molecular characterization

Sporangiospores were harvested from the infected leaves and genomic DNA was isolated using the traditional CTAB method. The concentration and purity of DNA were assessed using a nanodrop spectrophotometer (DeNovix, USA). PCR amplification was performed for two gene/regions: internal transcribed spacer one (ITS: ITS 6 and ITS 7), and beta-tubulin gene (TUB) using the universal primers (**Table 1**). After PCR amplification, the products were visualized on a 1% agarose gel under UV light using a Gel Doc™ XR+ Molecular Imager (Bio-Rad, USA) and amplicons were sequenced using commercial facility (Eurofins Genomics India Pvt. Ltd, Bangalore, India). The sequences were analyzed alongside related sequences from individual loci. Initial alignment was performed using the CLUSTAL algorithm within MEGA X (Tamura et al., 2021) with default parameters, followed by manual adjustments. Phylogenetic analyses was done using the concatenated sequences of ITS and β- tubulinin MEGA X (Tamura et al., 2021).

**Table 1:** Comparative analysis of *Plasmopara viticola* genomes available in NCBI.

Assembly metric	PV01 (India, this study)	INRA-PV221 (France)	JL-7-2 (China)	PvitFEM01 (Italy)
Assembly accession		GCA_001695595.3	GCA_001974925.1	GCA_003123765.1
Sequencing technology	Illumina + Oxford Nanopore	PacBio	Illumina HiSeq + PacBio	Illumina
Assembly level	Contig	Scaffold	Scaffold	Scaffold
Genome size (Mb)	84.09	92.90	101.30	83.50
Total ungapped length (Mb)	84.09	92.90	84.40	80.40
Number of scaffolds		358	2,165	57,890
Scaffold N50 (kb)		706.5	172.3	4.6
Number of contigs	182	374	23,193	65,120
Contig N50 (kb)	963.9	666.6	14.3	2.2
GC content (%)	44.61	45.0	45.0	44.0
Assembly coverage (×)		185×	207×	164×
Assembly method	Ragoo-polished hybrid	PBcR	AllPaths + PBJelly2	Ray

### Whole genome sequencing

High molecular weight genomic DNA was subjected to long-read sequencing using the Oxford Nanopore Technologies (ONT) platform. Library preparation was performed with the Ligation Sequencing Kit (SQK-LSK114, Chemistry v14) following the manufacturer’s protocol, including DNA repair, end-preparation, adapter ligation, and magnetic bead clean-ups. Native barcoding expansions (EXP-NBD114) were used for multiplexed libraries, while Rapid Barcoding Kits (SQK-RBK) were employed for low-input or pilot runs. Sequencing was conducted on MinION Mk1C or GridION devices, with PromethION utilized for high-throughput requirements. R9.4.1 flowcells were used for standard runs, whereas R10.x flowcells were preferred when improved homopolymer resolution was necessary. Basecalling was performed using Guppy in high-accuracy (sup) mode, and consensus polishing was carried out sequentially with Racon, Medaka, and Pilon using Illumina short reads for final correction. For Plasmopara genome sequencing (genome size ≈100 Mb), runs were planned to achieve 30–60× Nanopore coverage (~3–6 Gb per sample), based on historical flowcell yields and desired assembly depth.

Short-read sequencing was performed on Illumina platforms using Illumina DNA Prep (formerly Nextera DNA Flex) for whole-genome sequencing (WGS) and TruSeq DNA PCR-Free kits for high-quality, bias-minimized libraries when sufficient input DNA (>100 ng) was available. For Internal Transcribed Spacer (ITS) sequencing, dual-indexed amplicon libraries were prepared using a two-step PCR protocol and sequenced on the MiSeq system with v3 chemistry (2×300 bp) for near-complete ITS region coverage. Depending on project scale, WGS libraries were sequenced on MiSeq, NextSeq, or NovaSeq platforms employing Illumina’s sequencing-by-synthesis (SBS) chemistry with reversible terminators. Typical input DNA quantities ranged from 1–100 ng for DNA Prep and 100 ng for TruSeq PCR-Free protocols. A target of 50–100× Illumina coverage (2×150 or 2×250 bp reads; ~5–10 Gb data per 100 Mb genome) was maintained for hybrid assembly polishing, variant detection, and correction of Nanopore-derived assemblies.

### Raw data assessment

Quality control analysis of raw Illumina paired-end and nanopore reads performed using FastQC v0.11.9 (Andrews, 2010) and nanoQC (De Coster et al., 2018), respectively. Low-quality bases and adapter contaminations of paired-end were filtered using Fastp v.0.20.1(parameters: -f 10 -q 30) (Chen et al., 2018). Raw nanopore reads were filtered using NanoFilt (De Coster et al., 2018) (parameters: --head-crop 75 --tail-crop 60 Q 7) and post-filter quality assessment was done with FastQC and nanoQC, respectively. Error correction of the processed nanopore reads was done using NECAT (Chen et al., 2021).

### Genome assembly

Processed Illumina paired-end reads were initially assembled *de novo* using MEGAHIT v1.1.1 (Li et al., 2015a). A hybrid genome assembly was subsequently performed using MaSuRCA v3.2.2 (Zimin et al., 2013), incorporating both the processed Illumina reads and error-corrected Nanopore reads. Contigs generated from MEGAHIT and MaSuRCA were merged to produce a preliminary, redundant assembly. This merged assembly underwent redundancy reduction, gap filling, and scaffolding using the Redundans pipeline v0.14a (Pryszcz and Gabaldón, 2016).

The draft assembly was subjected to reference-guided scaffolding based on the Plasmopara viticola reference genome (RefSeq ID: GCA_001695595.3) using RaGOO v1.1 (Alonge et al., 2019). Eukaryotic contigs were identified and extracted from the RaGOO-scaffolded assembly using EukRep (West et al., 2018) to get the final contamination free draft assembly. Repetitive elements were subsequently masked with RepeatMasker v4.0.7 (Chen, 2004).

### Assessment of draft assembly

Assembly completeness was evaluated using BUSCO v5.0.0 (Simão et al., 2015), with assessments performed against the Alveolata_odb10 and Stramenopiles_odb10 lineage datasets using the parameters -m genome -l alveolata_odb10 and -m genome -l stramenopiles_odb10, respectively. Statistical assessment of the assembly was done using QUAST (Gurevich et al., 2013).

### ITSx based species identification

The final assembly was subjected to the ITSx v 1.1.3 (parameters: “--nhmmer”) to extract the ITS sequences. The extracted ITS sequences were submitted to online NCBI Megablast (McGinnis and Madden, 2004) (parameters: “Expect threshold: 0.000001, Organism: oomycetes (taxid:4762)) to verify the species.

### Draft genome annotation

The draft genome annotation was done using FunGAP (Min et al., 2017), that requires two inputs: genome assembly and RNA-seq reads and a protein sequence database. A total of 1 RNAseq sample of *P. viticola* was obtained from the NCBI BioProject PRJNA628826; https://www.ncbi.nlm.nih.gov/bioproject/?term=PRJNA628826). The forward and reverse reads of the individual samples were concatenated to generate one pair of forward (PRJNA628826_1.fq) and reverse (PRJNA628826_2.fq) reads. A non-redundant protein sequence database, required for (i) homology-based gene prediction by MAKER within FunGAP and (ii) evidence score calculation by FunGAP, was generated by downloading the closely related protein sequences of *P. viticola* (nr_prot.faa) using the download_sister_orgs.py utility of FunGAP. (parameters: -d sister_organisms -t *Plasmopara viticola* -n 100). Genome annotation using FunGAP was done based on the below mentioned parameters.

Three output files, gene features in GFF3, protein FASTA, and the annotation summary in HTML format were generated. The protein FASTA file was subjected to Diamond blastp v 2.0.6 (Buchfink et al., 2015) against NCBI’s non-redundant protein database (NRDB) against taxon “Oomycota” (parameters: --taxonlist 4762, -k 1 -f 5 -e 1e-5) and the tabular format of the xml file generated was obtained using Blast2GO (Conesa et al., 2005). The protein sequences were also provided to eggNOG-mapper (Huerta-Cepas et al., 2017) that provides Orthologous Groups (OGs) of proteins at different taxonomic levels, each with integrated and summarised functional annotations. Gene ontology (GO) annotation was done using PANNZER2 (Törönen et al., 2018). For KEGG annotation, the protein sequences were uploaded to KAAS KEGG Automatic Annotation Server (Moriya et al., 2007) to generate KO IDs (KEGG Orthology Identifier) based on best hits for each gene, which were further subjected to GAEV (Gene Annotation Easy Viewer) (Huynh and Xu, 2018) to summarise gene name, KO (KEGG Orthology) number, functional definition of ortholog and functional pathways that mapped to query genes.

### Secretome and effector prediction

Identification of secretory proteins in the proteome of *P.viticola* was done using the Predector pipeline (Jones et al., 2021). Predector runs numerous tools *viz*. SignalP (3, 4, 5, 6), TargetP (v2), DeepLoc (Almagro Armenteros et al., 2017), TMHMM (Krogh et al., 2001), Phobius (Käll et al., 2007), DeepSig (Savojardo et al., 2018), CAZyme finding (with dbCAN), Pfamscan, searches against PHI-base, Pepstats, ApoplastP, LOCALIZER, Deepredeff, and EffectorP 1, 2 and 3 for fungal secretome and effector discovery analysis, and outputs a list of ranked candidates. Prediction of effectors was done using an R package “effectR” (Tabima and Grünwald, 2018).

### Orthologous analysis

Orthologous gene clusters in *P. viticola* were identified using OrthoVenn3, an online tool for comparative analysis of orthologs (http://orthovenn3.bioinfotoolkits.net/). Gene sequences from *P. viticola* and other relevant species were uploaded as FASTA files, and pairwise comparisons were made to detect orthologs using BLAST-based algorithms. Venn diagrams were generated to visualize shared and unique gene clusters, and gene ontology (GO) enrichment analysis was performed to infer biological functions. Only genes with an e-value ≤ 1e-5 were retained, and the results were used to explore functional similarities and evolutionary relationships between *P. viticola* and other species.

### Transcriptomic analysis

#### Extraction of Total RNA, cDNA library construction, and illumina sequencing

Total RNA was extracted from grapes leaves (var. Bangalore blue) showing typical downy mildew symptoms caused by *P. viticola* and corresponding healthy controls using the Sigma Spectrum™ Plant Total RNA Kit following the manufacturer’s protocol. RNA quality and quantity were assessed using a NanoDrop 1000 spectrophotometer, Qubit™ 4 fluorometer with the RNA High Sensitivity Assay Kit, and Agilent 4150 Tapestation with High Sensitivity RNA ScreenTape. Samples with RIN ≥ 6 were used for library preparation using the TruSeq^®^ Stranded Total RNA Library Prep Kit (Illumina). Library concentration and insert size were measured using the Qubit DNA HS Assay Kit and Agilent Tapestation with High Sensitivity D1000 ScreenTape. Sequencing was performed on an Illumina NovaSeq 6000 platform to generate paired-end 150 bp reads with a depth of ~6 GB per sample.

#### Read preparation, alignment, and analysis of differentially expressed genes

FastQC v.0.11.9 (default settings) was used to examine the quality of the raw fastq readings from the sample (Andrews, 2010). Fastp v.0.20.1 was used to preprocess the raw fastq readings (Chen et al., 2018) with the following parameters: -trim_front1 8; -trim_front2 8; --length_required 50; -correction; -trim_poly_g; and -cut_mean_quality 30 and summarized using and MultiQC (Ewels et al., 2016). The processed reads were aligned to the *Vitis vinifera* (GCF_000003745.3) and *P. viticola* genome (PV01 Draft Assembly) using STAR aligner v 2.7.9a (Dobin et al., 2013) (parameters: outSAMtype BAM SortedByCoordinate -outSAMunmapped Within -quantMode TranscriptomeSAM, -outFilterScoreMinOverLread 0.33 -outFilterMatchNminOverLread 0.33). The rRNA and tRNA features were removed from the GTF annotation file of *V. vinifera* and *P. viticola*. The alignment file (sorted BAM) from individual samples was quantified using featureCounts v. 0.46.1 (Liao et al., 2014) based on the filtered GTF file to obtain transcript counts. These transcript counts for *V. vinifera* and *P. viticola* were used as inputs to edgeR with exactTest (Robinson et al., 2010) for differential expression estimation (parameters: dispersion = 0.1). An adjusted p-value threshold <= 0.05 and log2 fold change of ±1 was used for statistical estimation of gene expression. Gene ontology (GO) and Kyoto Encyclopedia of Genes and Genomes (KEGG) were performed on the ShinyGO (Ge et al., 2020) and iDEP 96 (Ge et al., 2018) enrichment tool based on the DAVID database to analyze and graphically represent the data.

### qPCR based validation

Total RNA was isolated from healthy and downy mildew infected grapevine leaves using the TRIzol reagent following the manufacturer’s instructions. First-strand cDNA was synthesized from 1 µg of total RNA using the Reverse Transcription Kit (Qiagen, Germany). Quantitative real-time PCR (qPCR) was performed on a CFX96 Real-Time PCR Detection System (Bio-Rad, USA) using gene-specific primers listed in **Supplementary File 1**. Relative gene expression levels were calculated using the 2^–ΔΔCt^ method, with the expression normalized against the actin gene.

## Results

### Morphology and phylogenetic identification of *Plasmopara viticola*

**Figure 1:**
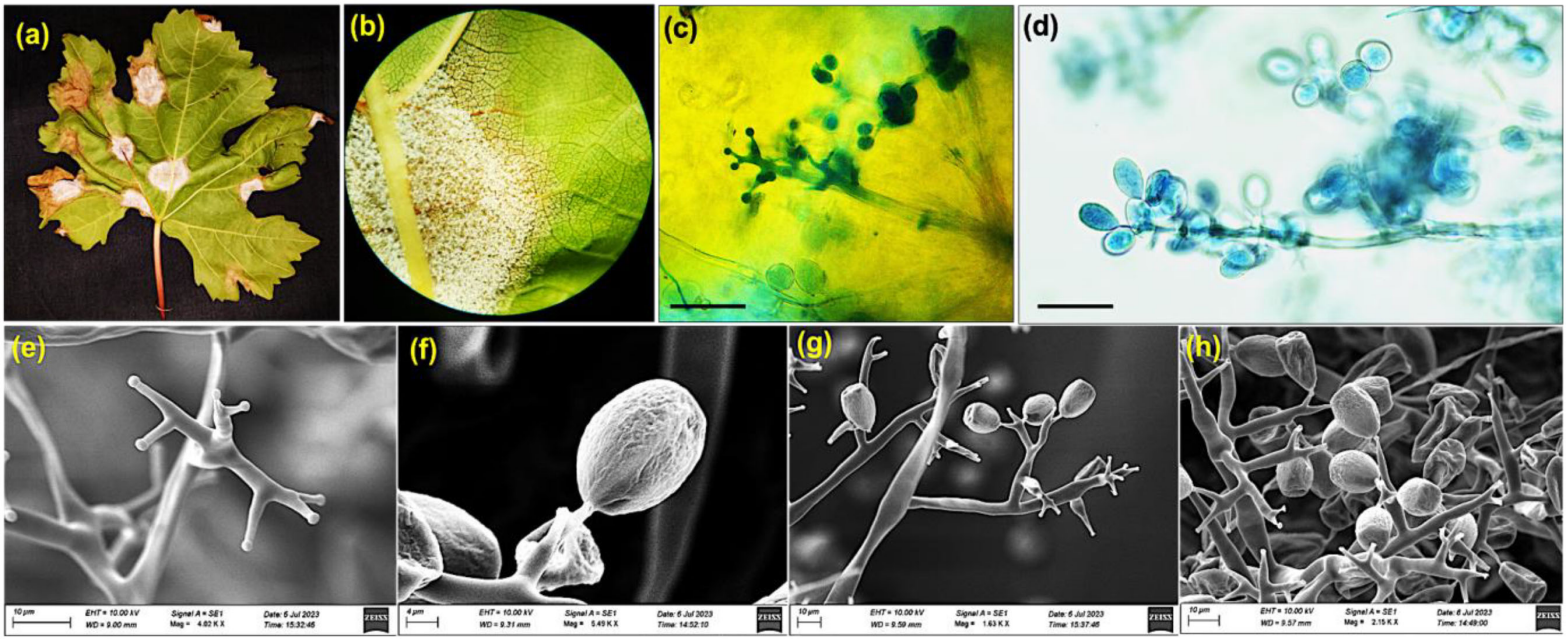
Morpho and microscopic characteristics of *P. viticola***(a)** A white downy fungal growth (sporangia) on the underside of the grape leaf, **(b)** Stereomicroscopic image of downy Mildew, **(c, d)** Microscopic image showing cross section of infected leaf tissue. SEM images of *P. viticola*, displaying **(e)** a right angle branched sporangiophore (Scale bar = 10μm), **(f)** characteristics features of single sporangia (Scale bar = 4μm), **(g, h)** sporangiophores bearing terminal, lemon-shaped sporangia (Scale bar = 10μm).

A total of twenty naturally infected grapevine leaf samples showing characteristic downy mildew symptoms were collected from major grapes growing regions of Karnataka. Infected leaves initially displayed small, greenish-yellow chlorotic spots on the adaxial surface, which gradually enlarged into irregular, oil-like lesions. On the abaxial surface, cottony white sporangiophores emerged under humid conditions typical of downy mildew infection (**Figures 1a, b**). Young berries exhibited browning, softening, and premature shattering, while infected shoots, tendrils, and petioles showed scalding, curling, and hook-shaped distortions. Floral tissues were highly susceptible, leading to rapid inflorescence withering, confirming the systemic colonization potential of *P. viticola* across all green organs of grapevine.

Microscopic examination of isolate PV01 revealed coenocytic, aseptate hyphae measuring 8–10 µm in diameter with frequent dichotomous branching. Sporangiophores were monopodial, with primary branches arising at right angles and bearing hyaline, lemon-shaped sporangia measuring 18.1 ± 1.3 × 13.8 ± 1.1 µm. Each sporangium released 1–10 biflagellate zoospores, which germinated within 15 minutes under moist conditions (**Figures 1c, d**). Scanning electron microscopy of the same isolate showed 3–6 primary and 2–4 secondary branches per sporangiophore, confirming structural features consistent with classical *P. viticola* descriptions (**Figures 1e,-h**).

**Figure 2:**
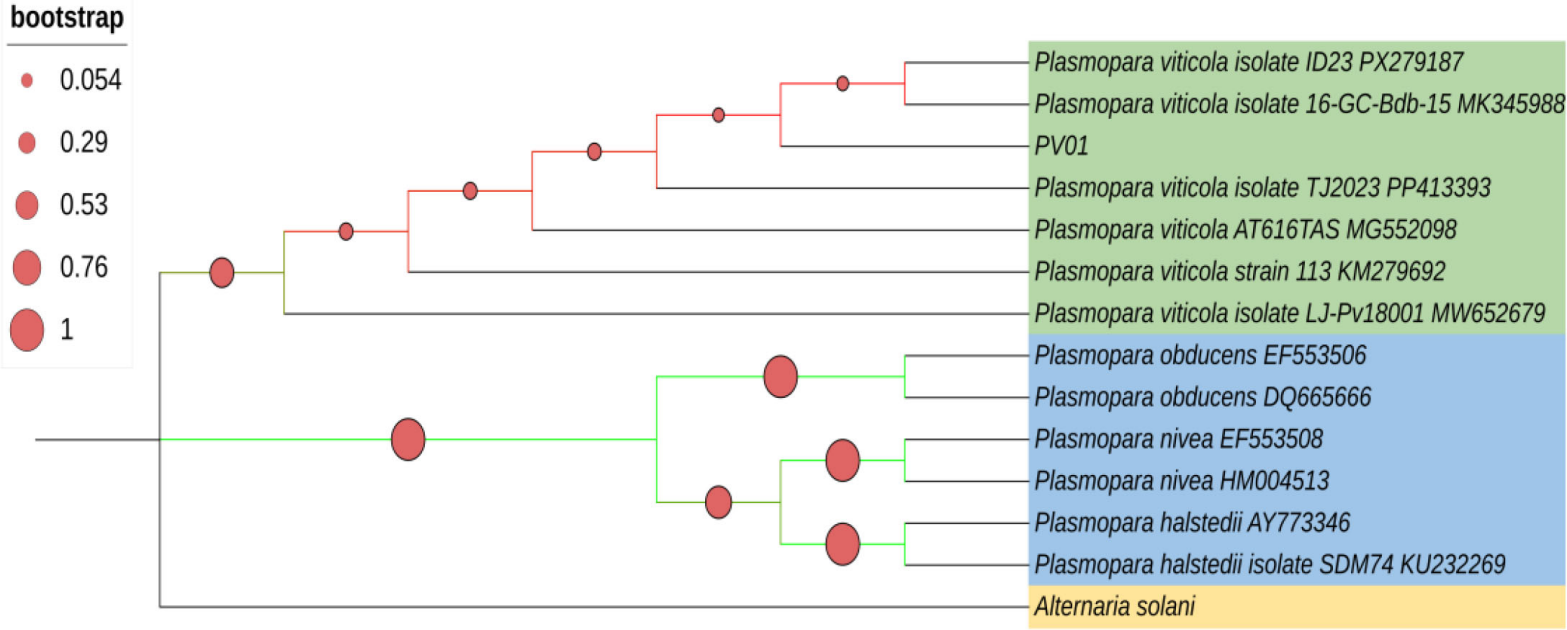
Neighbour-Joining phylogenetic tree constructed in MEGA 11 using ITS sequences of *Plasmopara* spp. Bootstrap support (1,000 replicates) is indicated by red circles at nodes, with circle size proportional to support values. The *P. viticola* isolate PV01 clusters with other *P. viticola* isolates and is clearly separated from related *Plasmopara* spp., while *Alternaria solani* was used as an outgroup. Lower bootstrap values within the *P. viticola* clade reflect low intraspecific divergence of the conserved ITS region.

For molecular identification, genomic DNA from the three representative isolates was amplified using ITS and β-tubulin gene regions, and the PV01 isolate was selected for phylogenetic analysis. Sequences are submitted in NCBI with the accession numbers of ITS (GenBank accession OK584068) and β-tubulin (GenBank accession ON637240). Phylogenetic analysis was performed to determine the taxonomic placement of the isolate PV01 within the genus *Plasmopara*. The phylogenetic tree clearly resolved species-level relationships, with high bootstrap support for nodes separating *P. viticola* from other *Plasmopara* species, including *P. halstedii*, *P. nivea*, and *P. obducens*. This robust interspecific support confirms the correct assignment of PV01 to *P. viticola*. Within the *P. viticola* clade, PV01 clustered together with other diverse *P. viticola* isolates, forming a monophyletic group. However, bootstrap support within this clade was comparatively low, indicating limited phylogenetic resolution among closely related *P. viticola* isolates (**Figure 2**).

The combined symptomatological, morphological, ultrastructural, and molecular evidence conclusively identified the isolate PV01 as *P. viticola*. This well-characterized isolate serves as a reference strain for ongoing genome and transcriptome analyses aimed at understanding effector diversity, host adaptation, and biotrophic evolution in *P. viticola* populations.

### Genome assembly and annotation

To elucidate the genomic architecture of the Indian *P. viticola* isolate PV01, a hybrid sequencing strategy combining Illumina paired-end short reads and Oxford Nanopore long reads was employed. This approach enabled both high base accuracy and long-range continuity, facilitating the resolution of repetitive and complex genomic regions. K-mer–based analysis of Illumina reads estimated a haploid genome size of approximately 75 Mb with a heterozygosity rate of ~1.2%, indicating moderate allelic diversity within the isolate. Repetitive elements accounted for ~13.1 Mb of the genome, while ~61.8 Mb comprised unique regions, consistent with genome features reported for obligate biotrophic oomycetes.

**Figure 3:**
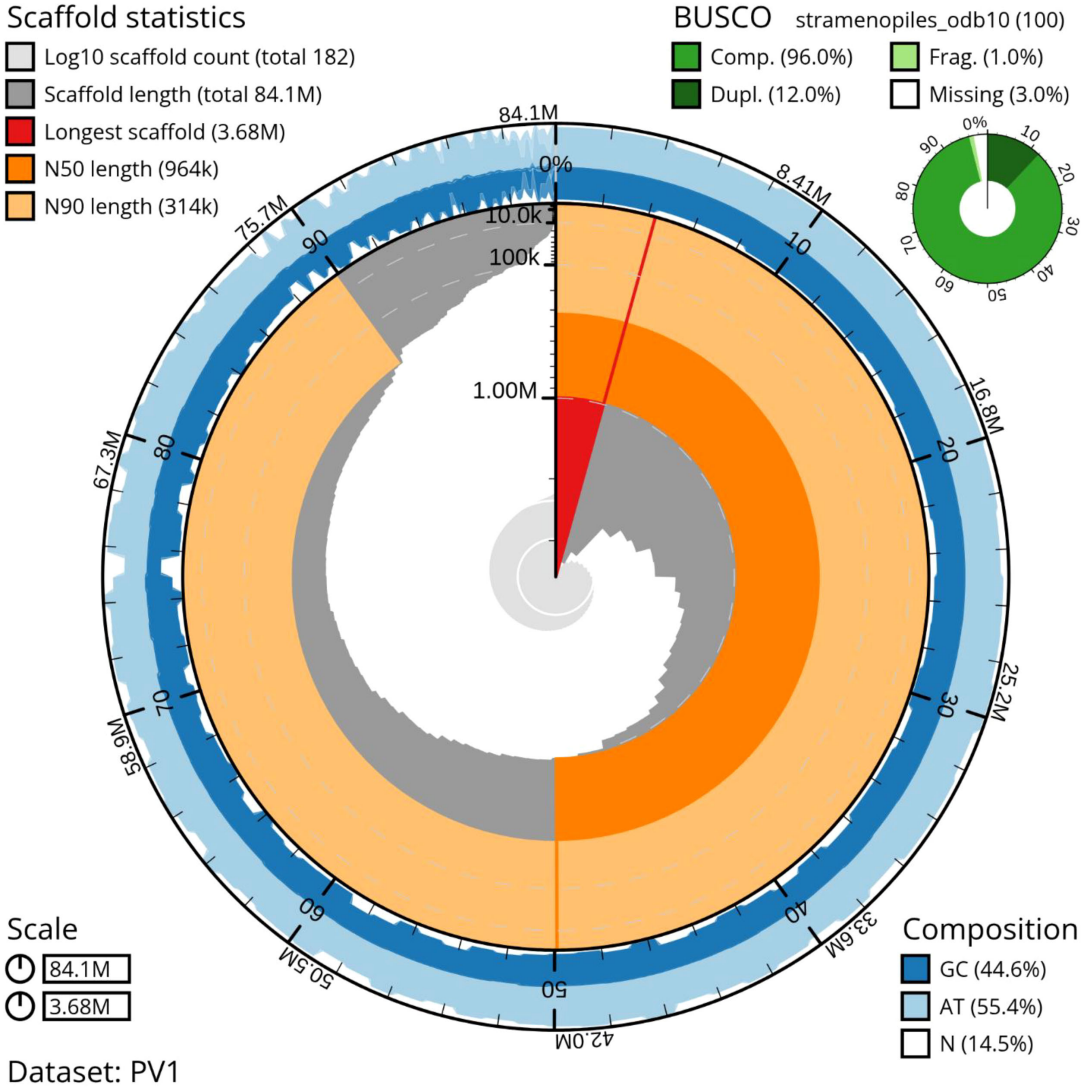
Snail plot showing genome assembly metrics of *Plasmopara viticola* isolate PV01. The plot summarizes assembly size (84.1 Mb), scaffold number (182), scaffold length distribution, N50/N90 values, and GC/AT/N composition. BUSCO analysis (stramenopiles_odb10) indicates high completeness (96.0%), supporting the quality of the assembly.

The final Ragoo-polished and eukaryote-filtered assembly comprised 182 contigs, spanning a total length of 84.09 Mb, with a contig N50 of 963.9 kb and an N90 of 314.1 kb. The largest contig measured 3.68 Mb, and the assembly exhibited a GC content of 44.61%. Assembly continuity was further supported by low fragmentation (L50 = 25) and a high auN value (1.29 Mb), reflecting uniform contig length distribution. Genome completeness was further evaluated using BUSCO v5.8.0 with the stramenopiles_odb10 dataset, recovering 97.0% complete BUSCOs, including 80.0% single-copy and 17.0% duplicated orthologs, with no fragmented BUSCOs and only 3.0% missing genes. The presence of duplicated BUSCOs is consistent with the diploid nature of P. viticola and genome heterozygosity. Together, the high BUSCO completeness, extensive transcriptome support, and broad functional annotation coverage demonstrate that the genome annotation is robust, comprehensive, and comparable in quality to previously published P. viticola genome resources, providing a reliable foundation for downstream functional, comparative, and evolutionary analyses (**Figure 3**).

Genome annotation of *P. viticola* was performed using the FunGAP pipeline, integrating genome assembly, RNA-seq evidence, and homology-based predictions, resulting in the identification of 12,404 protein-coding genes. The predicted gene models exhibited biologically consistent structural features, with average and median transcript lengths of 1,729.7 bp and 1,311.0 bp, respectively, and an average protein length of 500.8 amino acids (median: 375 aa). A substantial proportion of genes (7,943 genes; 64.04%) were spliced, with a median of two exons and two introns per gene, reflecting typical oomycete gene architecture. The genome showed a gene density of 147.17 genes per Mb.

Transcriptome-guided annotation was strongly supported by RNA-seq data, with 42.5 million reads mapped and assembled into 41,562 transcript contigs, including 27,404 contigs longer than 1 kb, representing a total assembled transcriptome size of 96.5 Mb. This extensive transcriptomic support indicates that a large fraction of predicted gene models are expressed and supported by experimental evidence.

Functional annotation of the predicted proteome demonstrated high coverage and biological relevance. Of the predicted proteins, 10,668 genes received functional annotations, with 8,177 proteins assigned putative functions based on orthology. Conserved protein domains were identified in 7,938 proteins, while 4,272 proteins were assigned KEGG orthologs, enabling pathway-level interpretation. In addition, 122 carbohydrate-active enzymes (CAZymes) were identified, consistent with the metabolic capabilities and host–pathogen interaction strategies of oomycete pathogens.

### Gene ontology annotation and functional classification

**Figure 4:**
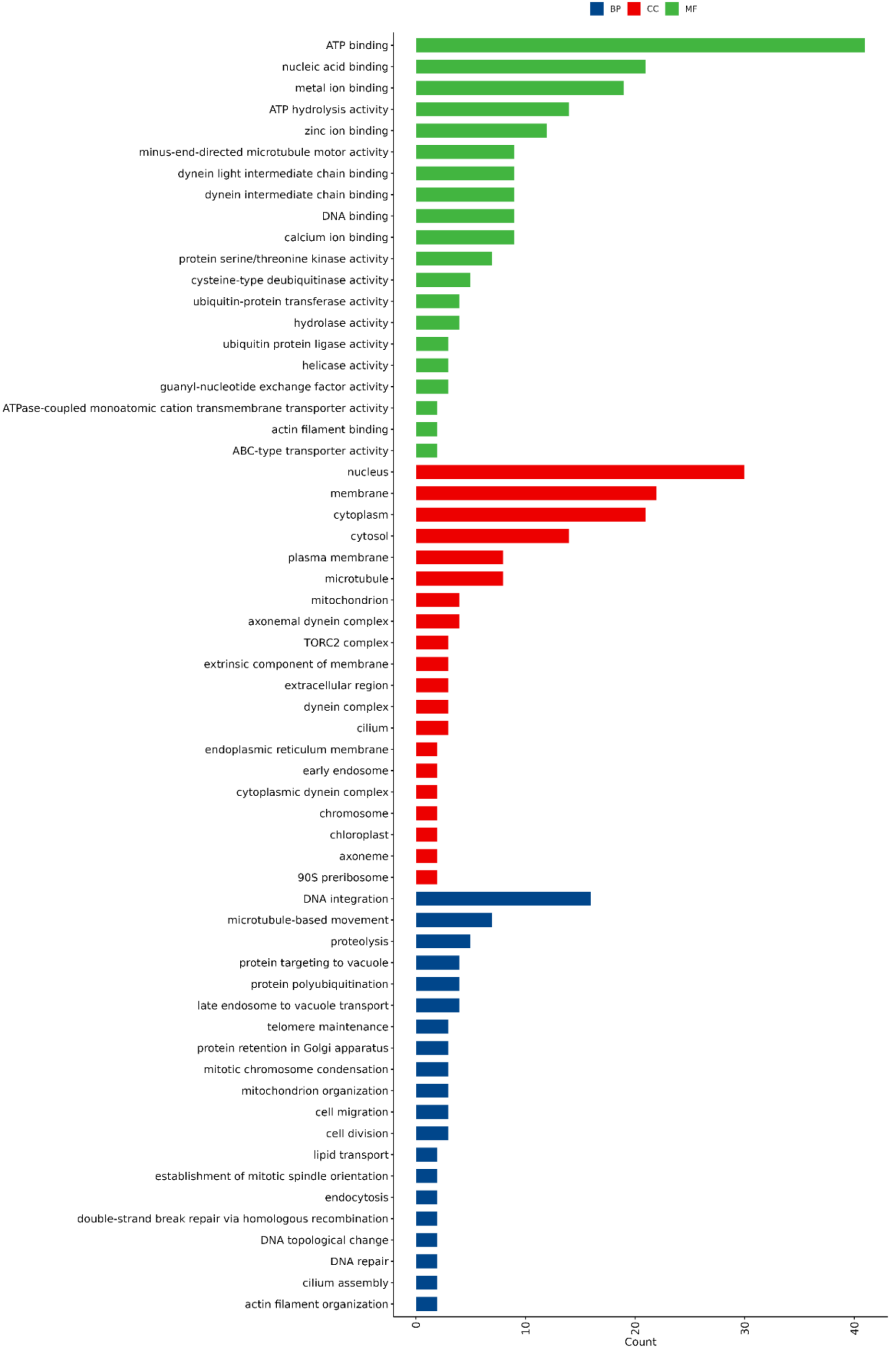
Gene Ontology (GO) classification of annotated genes in *Plasmopara viticola*. Genes are grouped into Biological Process (BP), Cellular Component (CC), and Molecular Function (MF) categories, showing the major functional classes represented in the genome.

Gene Ontology (GO) annotation of the *P. viticola* genome categorized the predicted genes into three major functional classes: Biological Process (BP), Cellular Component (CC), and Molecular Function (MF). Within the BP category, the most abundant terms were associated with DNA integration, proteolysis, protein targeting to the vacuole, and microtubule-based movement, indicating active genetic exchange, protein turnover, and cellular organization processes. The CC category was dominated by genes localized to the nucleus, cytoplasm, and membrane, followed by components such as mitochondrion, microtubule, and plasma membrane, suggesting the involvement of diverse intracellular and membrane-associated activities. In the MF category, ATP binding, nucleic acid binding, metal ion binding, and ATP hydrolysis activity were the most enriched terms, reflecting the metabolic and enzymatic versatility of the pathogen. Overall, the GO classification highlights that *P. viticola* possesses a complex and functionally diverse genome, supporting its adaptability and pathogenic lifestyle (**Figure 4**; **Supplementary File 2**).

### Comparative genomics and ortholog analysis

To place the PV01 genome in a comparative context, we assessed its assembly features against previously published *P. viticola* genomes from Europe and China (**Table 1**). The French isolate INRA-PV221, generated using PacBio sequencing, consists of 374 contigs with a contig N50 of 666.6 kb, whereas the Chinese isolate JL-7–2 and the Italian isolate PvitFEM01 are highly fragmented, comprising 23,193 contigs (N50: 14.3 kb) and 65,120 contigs (N50: 2.2 kb), respectively. Despite comparable genome sizes ranging from 83 to 101 Mb and similar GC content (~44–45%) across all isolates, the PV01 assembly exhibits the lowest contig number and the highest contig N50 among the currently available *P. viticola* genomes.

**Figure 5:**
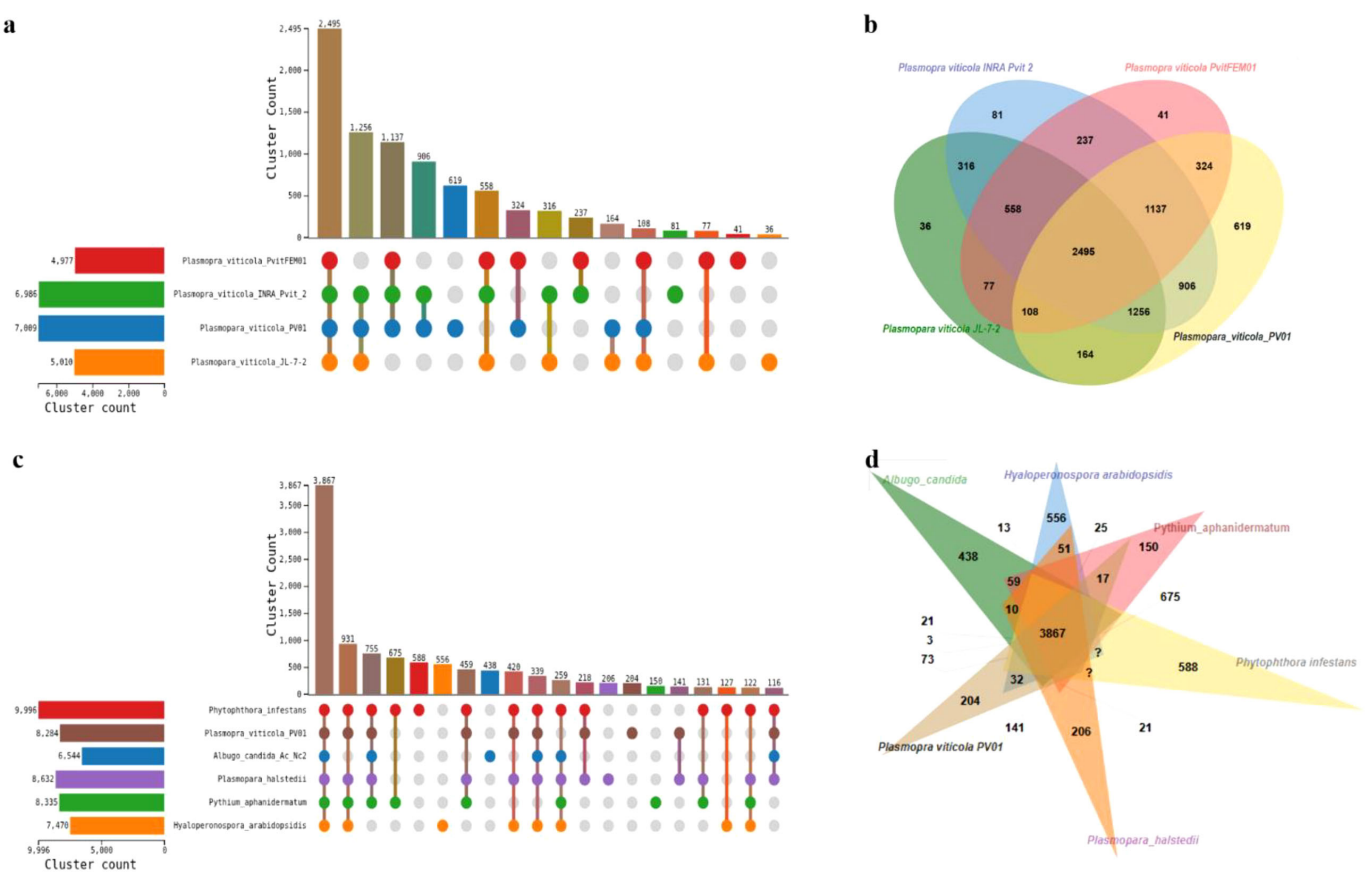
Comparative genomic analysis of *Plasmopara viticola* PV01. **(a)** UpSet plot showing shared and unique orthologous gene clusters among *P. viticola* PV01 and other *P. viticola* genomes. **(b)** Venn diagram illustrating the distribution of orthologous proteins among *P. viticola* genomes. **(c)** UpSet plot showing shared and unique orthologous gene clusters between *P. viticola* PV01 and other oomycete genomes. **(d)** Venn diagram depicting orthologous protein distribution between *P. viticola* PV01 and representative oomycetes.

Orthologous gene clustering among four *P. viticola* isolates (PV01, JL-7-2, INRA_Pvit_2, and PvitFEM01) was examined using UpSet analysis. A total of 2,495 orthogroups were shared by all four isolates, comprising 13,915 proteins. Major partial intersections included 1,256 orthogroups shared by PV01, JL-7-2, and INRA_Pvit_2 (5,137 proteins), 1,137 orthogroups shared by PV01, INRA_Pvit_2, and PvitFEM01 (4,637 proteins), 906 orthogroups shared by PV01 and INRA_Pvit_2 (2,533 proteins), and 619 orthogroups shared by PV01 and JL-7-2 (1,638 proteins). Isolate-specific orthogroups numbered 164 in PV01, 108 in JL-7-2, 81 in INRA_Pvit_2, and 77 in PvitFEM01, with additional smaller intersections ranging from 41 to 36 orthogroups and corresponding protein counts (**Figures 5a, b**).

To further contextualize orthogroup conservation at the interspecific level, ortholog clustering was extended to six oomycete species, including *Albugo candida*, *Hyaloperonospora arabidopsidis*, *Pythium aphanidermatum*, *Phytophthora infestans*, *Plasmopara halstedii*, and *P. viticola* PV01. This analysis identified 3,867 orthogroups shared across all six species, representing the largest intersection and comprising 30,498 proteins. Additional prominent intersections included 931 orthogroups shared among *P. infestans*, *P. viticola* PV01, *P. halstedii*, and *H. arabidopsidis* (6,245 proteins), 755 orthogroups shared among *A. candida*, *P. infestans*, *P. viticola* PV01, and *P. halstedii* (4,833 proteins), and 675 orthogroups shared between *P. infestans* and *P. viticola* PV01 (2,137 proteins). Species-specific orthogroups comprised 588 in *P. infestans*, 556 in *H. arabidopsidis*, and 438 in *A. candida*, with the remaining intersections ranging from 459 to 116 orthogroups (**Figures 5c, d**).

### Genome architecture of PV01

**Figure 6:**
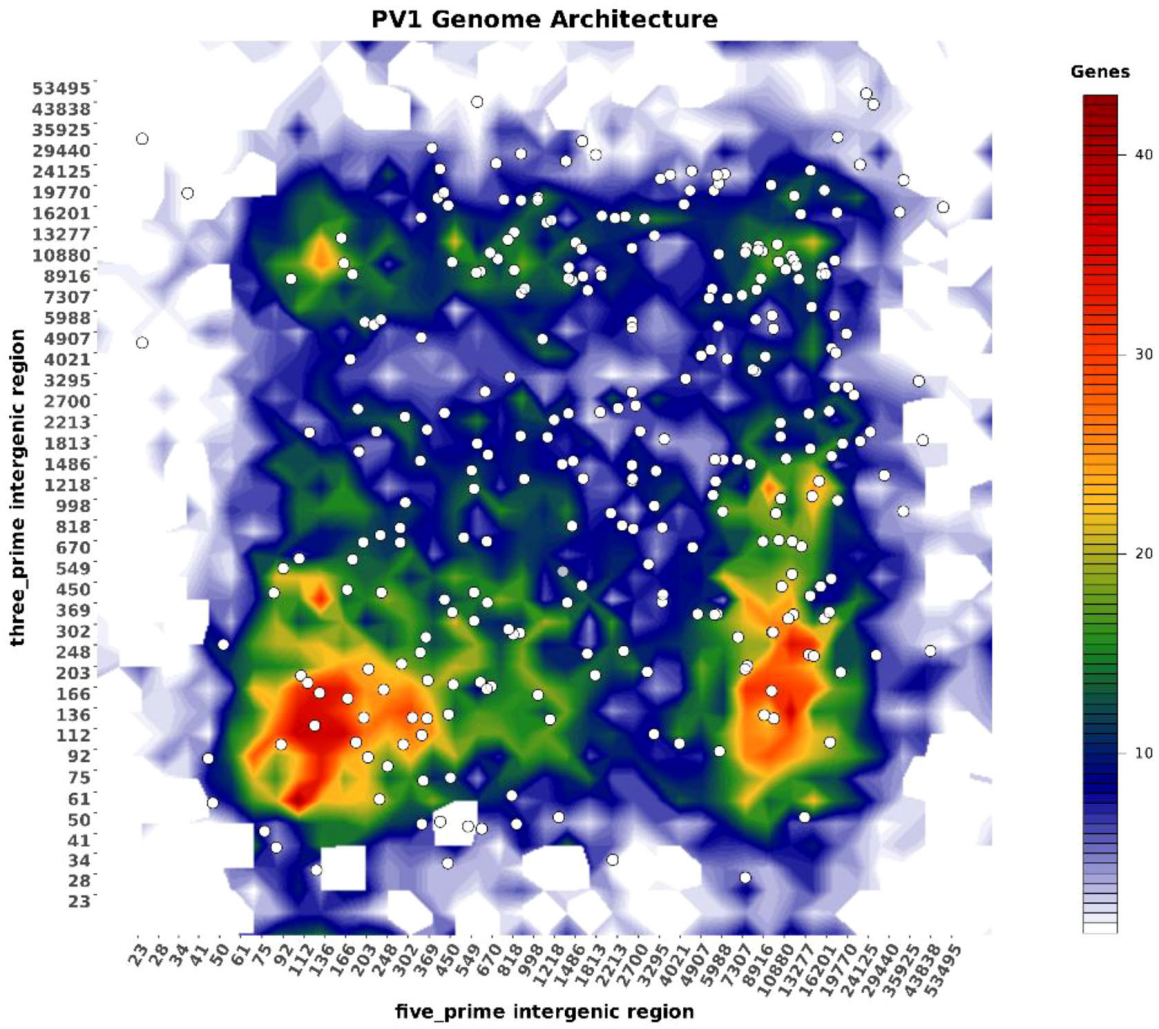
Two-dimensional density plot illustrating the genome architecture of *Plasmopara viticola* isolate PV01. The plot shows the distribution of genes based on 5′ and 3′ intergenic distances, with color intensity representing local gene density. Gene-rich, compact regions are shown in blue–green, whereas gene-sparse regions with larger intergenic distances appear in red–orange, highlighting a characteristic two-speed genome organization.

To rigorously assess genome compartmentalization in the Indian *P. viticola* isolate PV01, we quantified gene density and intergenic distance distributions using the final curated gene annotation. For each predicted gene, upstream (5′) and downstream (3′) intergenic distances were calculated and jointly analyzed to capture local genomic spacing patterns. Intergenic distances exhibited pronounced heterogeneity across the genome. The median five-prime intergenic distance was 963 bp, with an interquartile range of 205–6,957 bp, while the median three-prime intergenic distance was 454 bp (interquartile range: 138–3,590 bp). Notably, the upper tails of these distributions were extensive, with the 90th percentiles reaching ~13.8 kb (5′) and ~11.6 kb (3′), indicating the presence of large gene-free intervals. Based on these distributions, genes flanked by intergenic regions ≥5 kb on at least one side were classified as residing within gene-sparse regions (GSRs). Using this criterion, 46.2% of predicted genes were localized to GSRs, while the remaining genes were positioned within gene-dense regions (GDRs) characterized by short flanking intergenic distances. This near-equal partitioning of genes between compact and expanded genomic compartments highlights a strongly bipartite genome organization. Two-dimensional density analysis of five-prime versus three-prime intergenic distances further emphasized this structure, revealing distinct clusters corresponding to GDRs and GSRs (**Figure 6**). Such spatial segregation of genes is a defining feature of two-speed genomes described in oomycetes and other filamentous pathogens, where gene-sparse, repeat-rich regions are often associated with enhanced structural plasticity and adaptive potential (**Figure 6**).

### Secretome and effector repertoire of *P. viticola*

A comprehensive in silico pipeline integrating SignalP 6.0, EffectorP 3.0, and EffectorDB was used to predict secretory and effector proteins in the *P. viticola* proteome. Among the 12,404 predicted protein-coding genes, SignalP identified 988 proteins carrying N-terminal signal peptides, indicating their potential secretion through the classical secretory pathway. EffectorP analysis predicted 1,615 proteins as putative effectors based on machine learning–derived effector characteristics. Integration of these predictions identified 988 high-confidence secreted effector candidates, representing proteins that are both secreted and effector-like.

Homology-based searches against EffectorDB further identified 1,615 proteins matching known effector families, encompassing 155 distinct effector domains, including well-characterized motifs such as RXLR, CRN (Crinkler), and Nep1-like proteins (NLPs). These results indicate that *P. viticola* encodes a diverse and extensive effector repertoire consistent with its biotrophic lifestyle (**Supplementary Files 3**, **4**).

### Functional composition of the secretome and virulence-associated proteins

**Figure 7:**
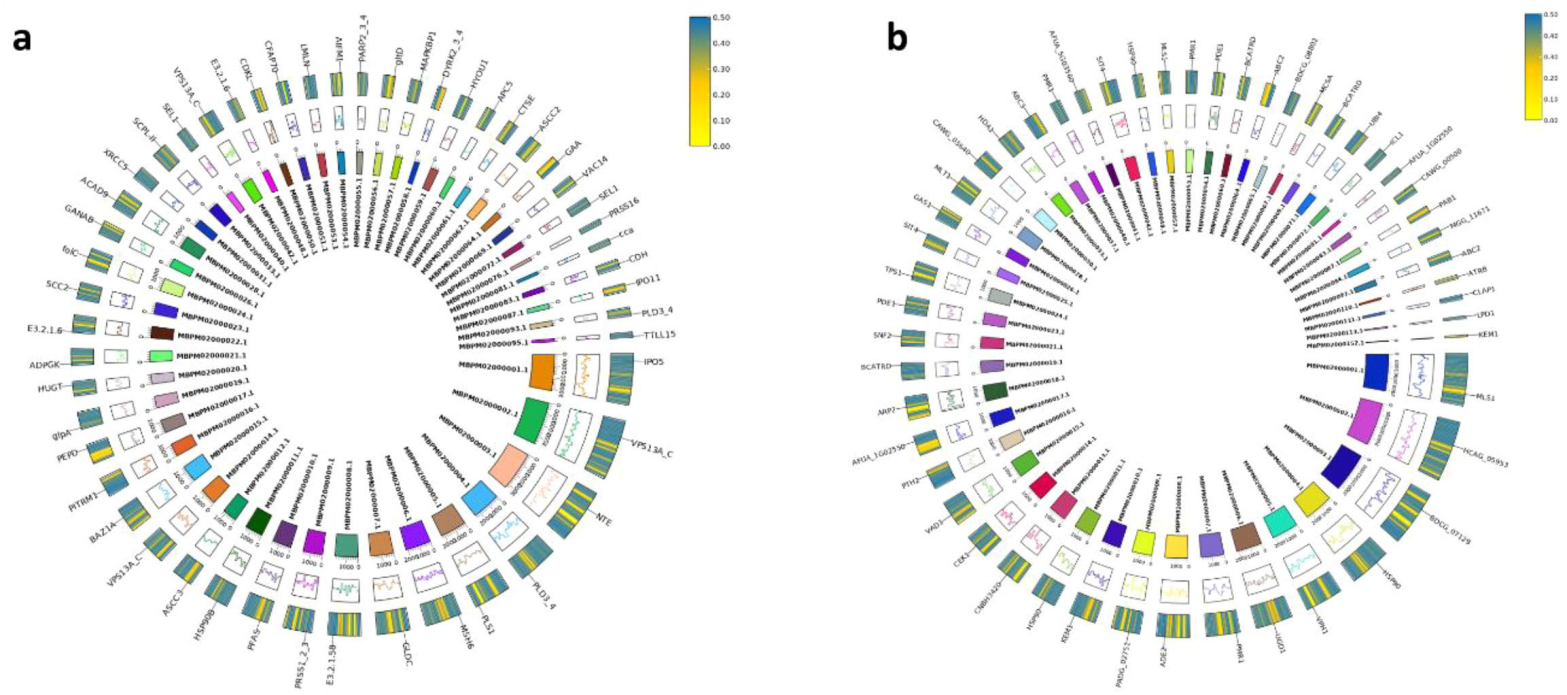
Circos plots of *Plasmopara viticola* PV01 showing genomic distribution of predicted pathogenicity-related genes. **(a)** Secreted proteins identified by DIAMOND BLASTX against a secretome database. **(b)** Virulence-associated genes identified by DIAMOND BLASTX against virulence factor databases. Colored tracks represent individual scaffolds, and bars indicate the genomic positions and relative abundance of matched genes across the genome.

Detailed analysis of the predicted secretome identified 50 proteins with high similarity to experimentally characterized secretory proteins, including hydrolytic enzymes such as endo-1,3(4)-β-glucanase, lysosomal α-glucosidase, and trypsin-like proteases, which are likely involved in host cell wall degradation and nutrient acquisition. Additional secreted components included transport-related proteins (e.g., IPO5, IPO11, VPS13A/C), metabolic enzymes (e.g., UDP-glucosyltransferases), and chaperones such as HSP90B, reflecting a multifunctional toolkit for host–pathogen interactions (**Figure 7a**).

Parallel genome-wide screening identified 50 high-confidence virulence-associated genes with strong homology to known fungal virulence determinants. These included ATP-binding cassette (ABC) transporters, P-type ATPases, and carnitine acetyltransferases, which are implicated in detoxification and metabolic adaptation. Stress-responsive proteins such as HSP90 and DEAD-box RNA helicases (e.g., VAD1) were also detected, along with metabolic enzymes and regulatory proteins (e.g., malate synthase, trehalose-6-phosphate synthase, UDP-glucose dehydrogenase, SNF2, CEK1), highlighting a multifaceted virulence strategy (**Figure 7b**).

### RXLR and CRN effector families

Motif-based analysis revealed a limited number of canonical RxLR effectors, with four proteins containing the RxLR motif but lacking the EER motif. Additionally, six proteins encoded the EER motif alone, suggesting divergence from classical RxLR effector architecture, while 52 candidates lacked recognizable RxLR/EER motifs, indicating the presence of non-canonical effectors (**Supplementary File 5**).

In contrast, CRN (Crinkler) effectors were highly expanded with 1,198 CRN proteins identified. These proteins displayed conserved LXLFLAK and DWL (HVLVVVP) domains characteristic of CRN architecture. Given that CRN effectors are often associated with host cell death modulation, their marked expansion suggests a central role in *P. viticola* pathogenicity (**Supplementary File 6**).

### Host-pathogen transcriptomic dynamics

#### Grapevine transcriptomic response

**Figure 8:**
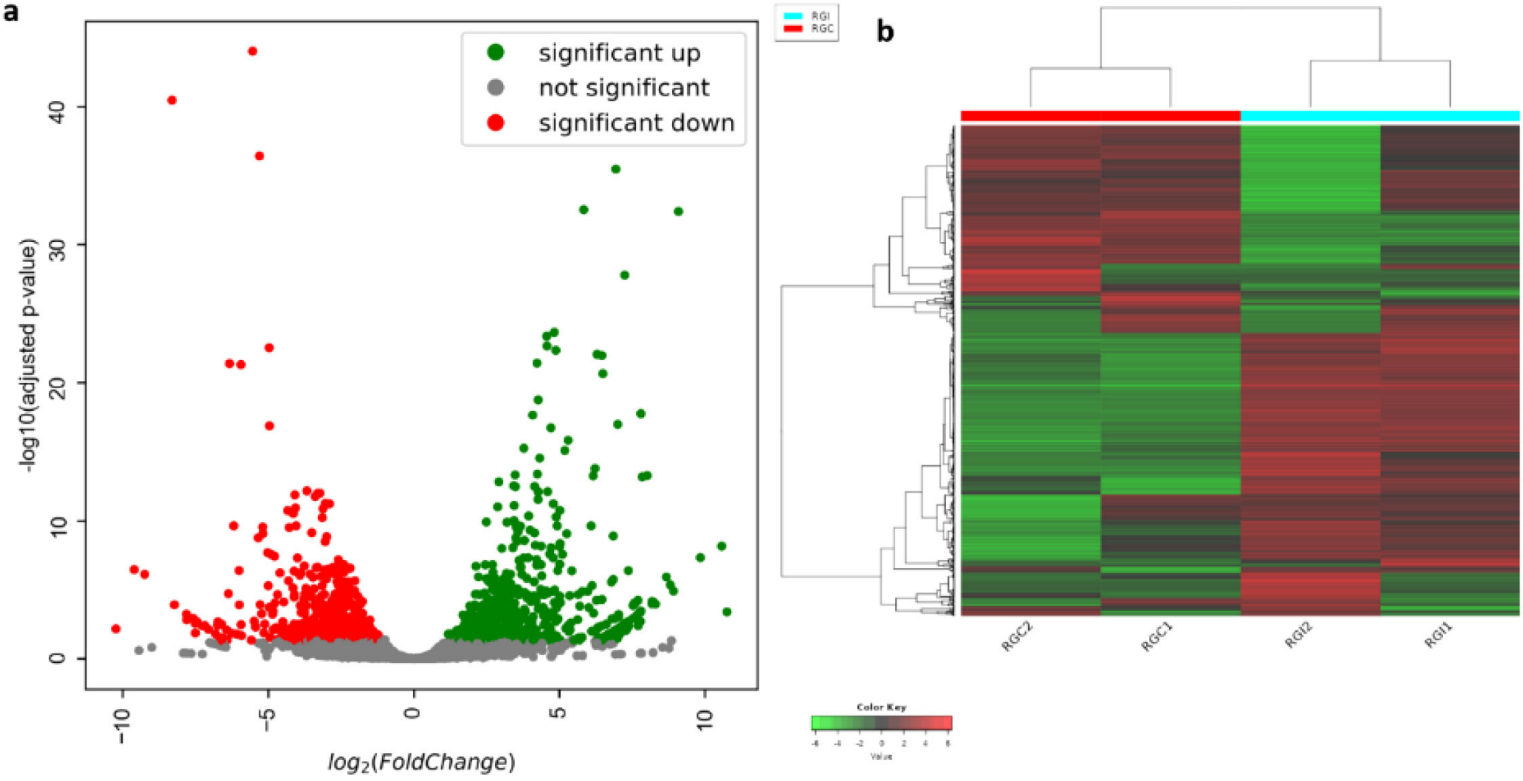
Differential gene expression analysis of grape samples infected with downy mildew. **(a)** Volcano plot showing differentially expressed genes (DEGs), with upregulated genes in green, downregulated genes in red, and non-significant genes in grey based on log_2_ fold change and adjusted p-value thresholds. **(b)** Heatmap illustrating hierarchical clustering and expression patterns of DEGs across control and infected samples.

Dual RNA-Seq analysis of infected grapevine leaves enabled simultaneous profiling of *V. vinifera* and *P. viticola* transcripts at infection sites. Reads were mapped to the combined reference genomes of *V. vinifera* (GCF_000003745.3) and *P. viticola* (PV01 Draft Assembly). Using thresholds of p ≤ 0.05 and log2 fold change ≥ 1, a total of 2,061 differentially expressed genes (DEGs) were identified in *V. vinifera*, with 1,384 upregulated and 1,040 downregulated compared to uninfected controls (**Figure 8a**).

Defense-related genes were strongly induced. DEGs included those encoding jasmonic acid and ethylene signaling enzymes, as well as phytoalexin biosynthesis enzymes such as Flavanone 3-hydroxylase (F3H, XM_002285694.4). Leucine-rich repeat receptor-like kinase (RLK, XM_002276089.4) expression was also increased, consistent with activation of pattern recognition receptors (PRRs) and receptor-like serine/threonine kinases (STKs). Upregulation of MAPK2 (XM_010657859.2) indicated activation of MAPK signaling cascades. Calcium/calmodulin (CaM/CML) transcripts (XM_002274440.4, XM_002283534.4) were also upregulated. Several WRKY family transcription factors (NM_001280990.1, XM_002262739.4, XM_002272468.3, XM_002272684.3, XM_002279371.4) were induced, alongside multiple pathogenesis-related (PR) proteins: PR-1 (XM_002276831.3, XM_002273752.3, XM_002274069.4), PR-4 (XM_002264684.4, XM_002264611.4), PR-5 (NM_001281202.1, XM_010662681.2), PR-9 (XM_002267758.3, XM_002269136.4, XM_002269882.4, XM_002274733.3, XM_002275252.3), and PR-10 (XM_002274072.4). Detoxification-related genes such as glutathione S-transferase (GST, XM_002285176.4) and cytochrome P450s (NM_001281186.1, XM_002262657.3) were also activated (**Figure 8b**; **Supplementary File 7**).

### GO enrichment analysis

**Figure 9:**
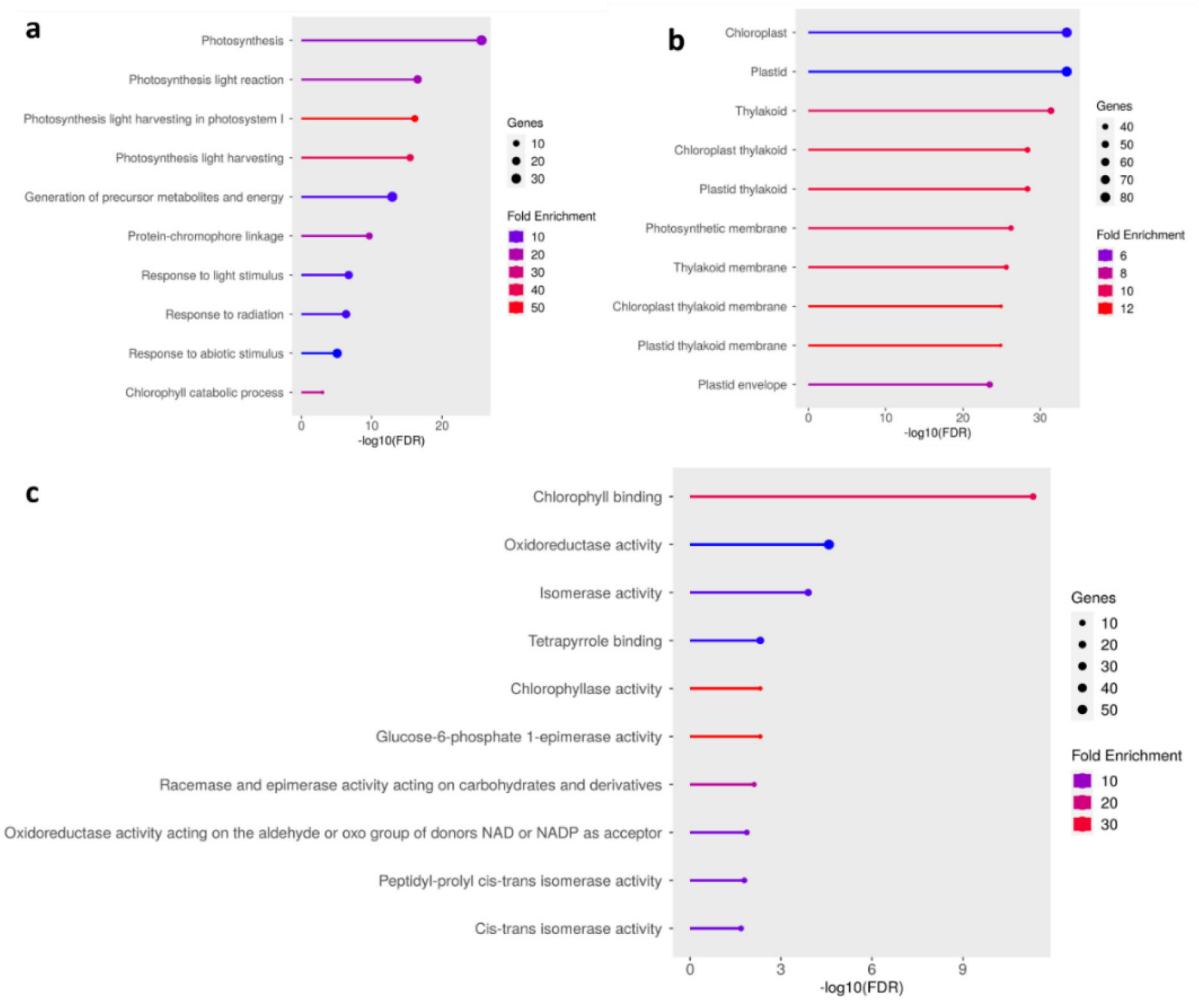
Gene Ontology (GO) enrichment analysis of differentially expressed genes (DEGs). The top enriched GO terms are shown for **(a)** Biological Process, **(b)** Cellular Component, and **(c)** Molecular Function categories. The x-axis represents −log10(FDR), dot size indicates the number of genes, and color denotes fold enrichment.

GO enrichment analysis revealed that the most significantly enriched cellular components confirmed that most downregulated DEGs were associated with chloroplast structures, including chloroplasts, plastids, thylakoids, photosynthetic membranes and thylakoid membranes (**Figure 9a**). In the molecular function category, chlorophyll binding was the most enriched term, followed by oxidoreductase activity, isomerase activity, and tetrapyrrole binding. Specific enzymatic functions such as chlorophyllase activity, racemase/epimerase activity, and peptidyl-prolyl cis-trans isomerase activity were also affected, reflecting a targeted repression of pigment-binding and redox-active enzymes (**Figure 9b**). Biological processes were photosynthesis and light responses, which were strongly downregulated. Suppressed pathways included photosynthesis, photosynthetic light reactions, photosystem I light harvesting, and the generation of precursor metabolites and energy. Additional enrichment of response to light stimulus, response to radiation, and chlorophyll catabolic process indicated broad repression of chloroplast function under pathogen stress (**Figure 9c**).

### Enrichment pathways

**Figure 10:**
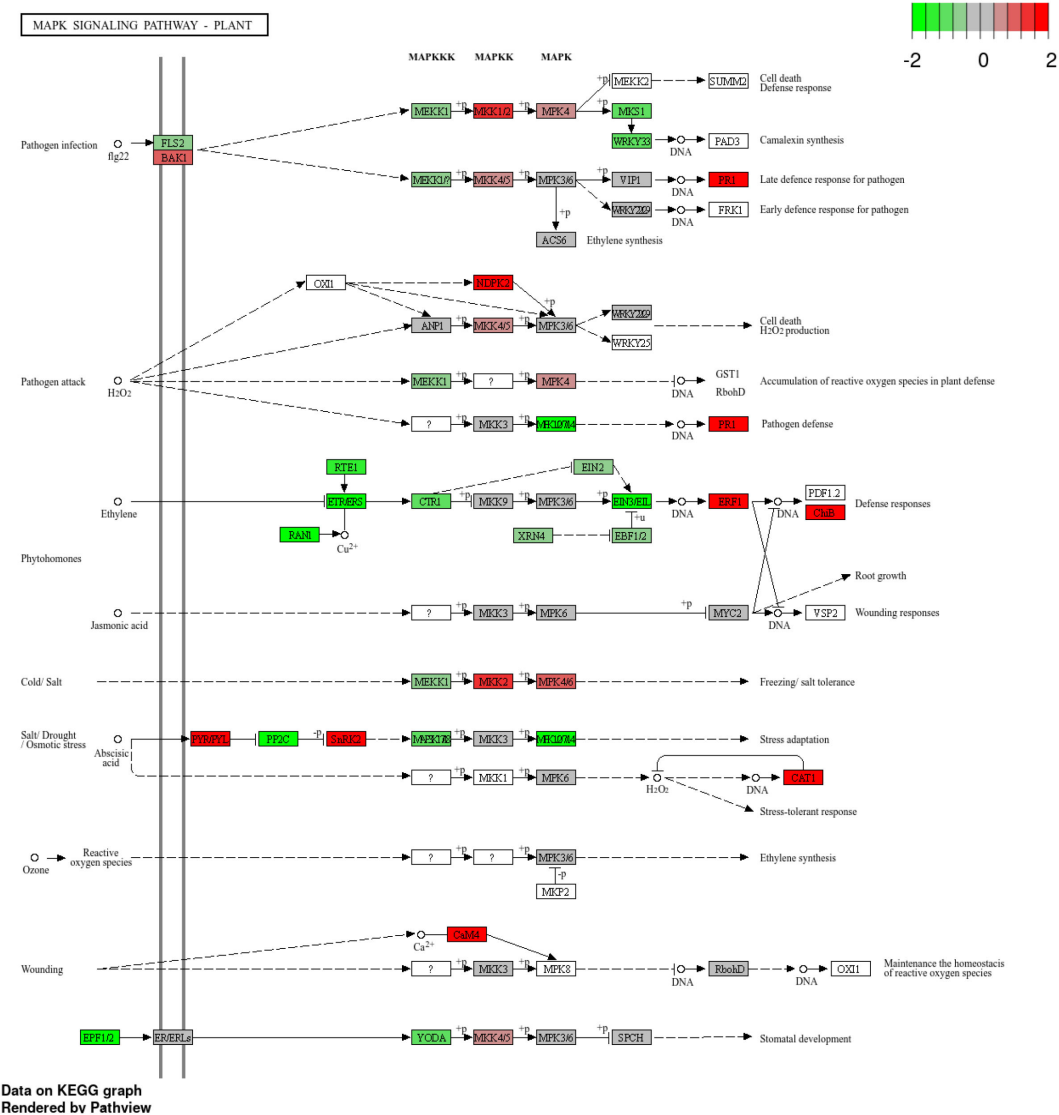
KEGG MAPK signaling pathway showing enriched genes in grape during downy mildew infection. Differentially expressed genes are mapped onto the plant MAPK pathway, with red indicating upregulated and green indicating downregulated genes (log_2_ fold change scale shown).

Key immune signaling pathways were engaged. In the plant–pathogen interaction pathway, genes such as FLS2, EFR, CERK1, RPM1, RPS2, PBS1, RBOH, WRKY33, and PR1 were upregulated, supporting activation of both PTI and ETI responses. Negative regulators such as CPKs, CNGCs, and RIN4 were downregulated, favoring defense activation. In the MAPK signaling pathway, MAPKKK17/18, MPK3, and WRKY33 were induced, together with transcription factors MYC2 and EIN3, consistent with hormone signaling via jasmonic acid and ethylene (**Figure 10**).

**Figure 11:**
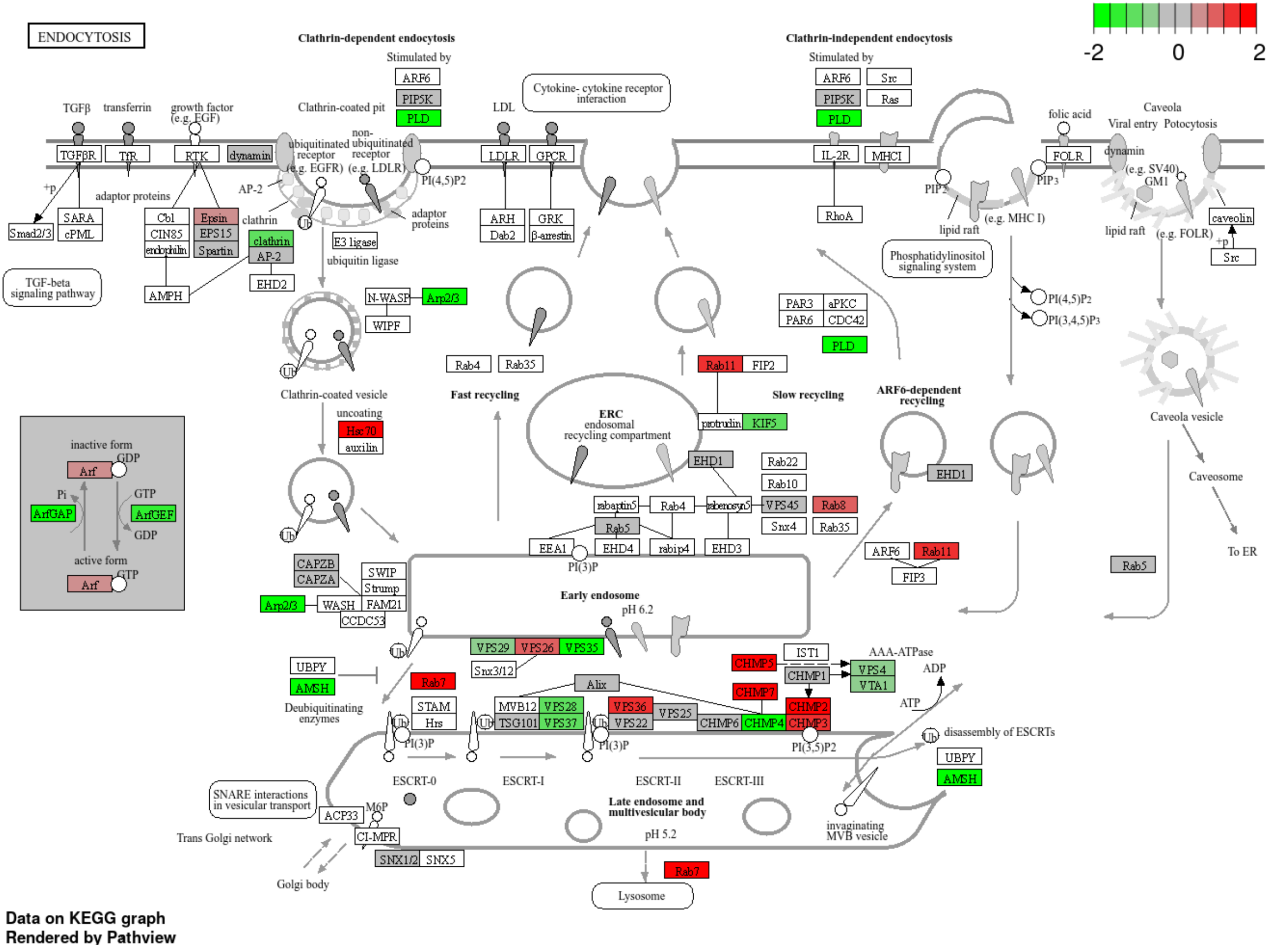
KEGG endocytosis pathway showing enriched genes in grape during downy mildew infection. Differentially expressed genes are mapped onto the endocytosis pathway, with red indicating upregulated and green indicating downregulated genes (log_2_ fold change scale shown), highlighting the involvement of vesicle trafficking and membrane dynamics in the host response to infection.

**Figure 12:**
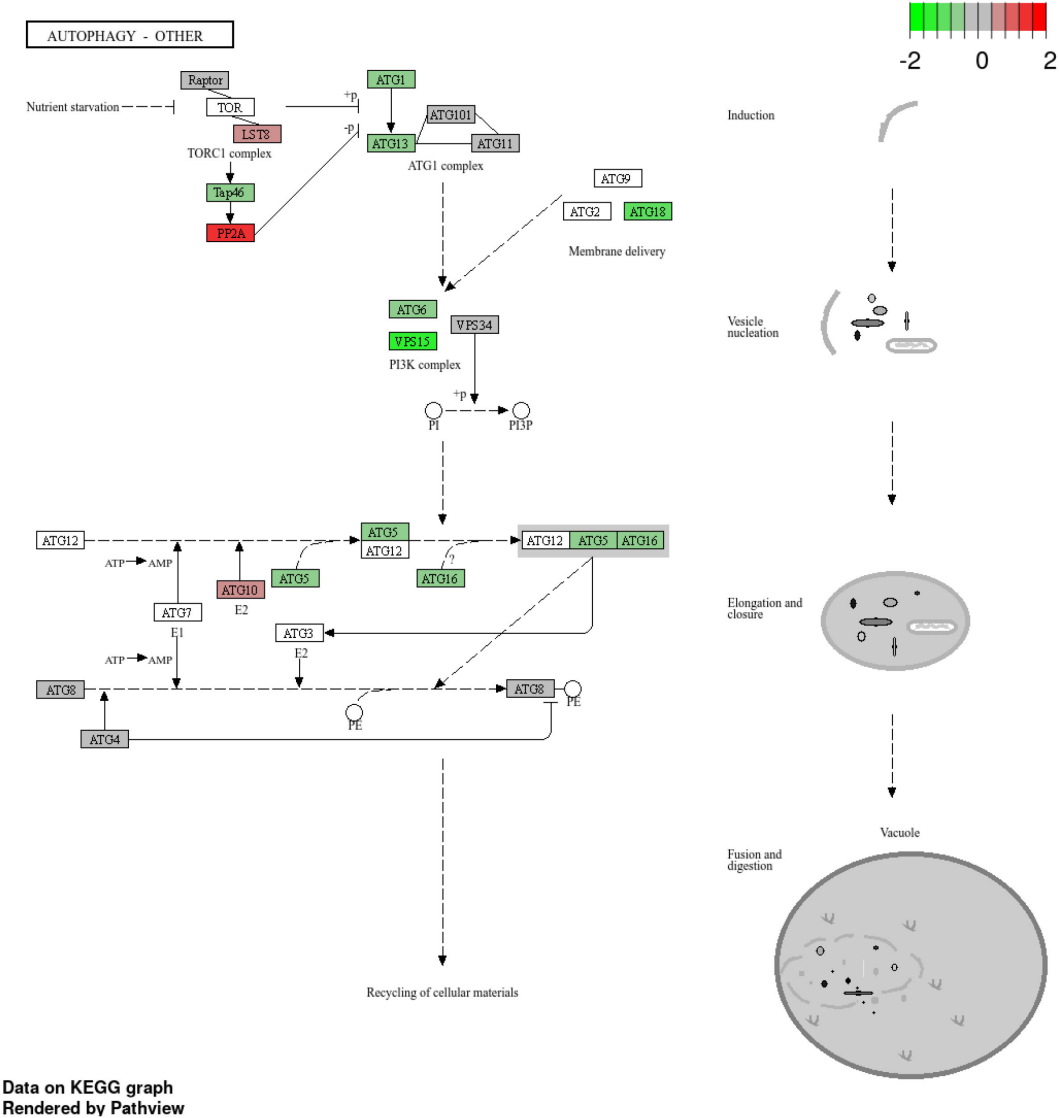
KEGG autophagy pathway showing enriched genes in grape during downy mildew infection. Differentially expressed genes are mapped onto the autophagy pathway, with red indicating upregulated and green indicating downregulated genes (log_2_ fold change scale shown), highlighting the role of autophagy-related processes in the host defense response.

Transcriptome profiling also revealed strong modulation of vesicle trafficking, phagosome, endocytosis, and autophagy pathways. Genes encoding Rab7, TUBA, TUBB, RILP, Dynein, vATPase, CALR (calreticulin), Sec61, and Rac were upregulated, indicating enhanced vesicle transport, ER-mediated processing, and ROS generation. Conversely, regulators of endosomal maturation such as PIKFYVE, CHMP5, CHMP1A, ArfGAPs, and PLD were downregulated (**Figure 11**). Autophagy-related genes, including ATG1, ATG5, ATG6, ATG12, ATG13, ATG16, ATG18, ATG9, and VPS15, were consistently suppressed, reflecting inhibition of host autophagy flux (**Figure 12**).

### Pathogen transcriptomic response

A prominent feature of the PV01 transcriptome was the strong induction of apoplastic effector candidates, particularly trypsin-like serine proteases (e.g., gene_02080, gene_00705, gene_02140, gene_02167, gene_02299), which showed high infection-specific expression. These proteases are predicted to function in the host apoplast and are implicated in modification or suppression of host defense proteins without triggering host cell death, consistent with a biotrophic infection strategy. Notably, glucanase inhibitor proteins (gene_02559 and gene_02596) were among the most highly induced pathogen genes. These proteins are known to inhibit host β-glucanases and likely contribute to suppression of cell wall–derived defense responses, thereby facilitating sustained colonization of living host tissue.

Although canonical RXLR or CRN effector motifs were not broadly annotated, an RXLR-like effector candidate (gene_02654) displayed strong infection-specific induction, suggesting the presence of divergent cytoplasmic effectors in the PV01 isolate. In addition, multiple genes encoding components of the ubiquitin and SUMO-like modification machinery (gene_10618, gene_02018, gene_03440, gene_03495) were strongly upregulated, indicating potential manipulation of host protein stability and immune signaling through post-translational regulation. PV01 also induced a selective set of carbohydrate-active enzymes (CAZymes) and cell wall–modifying enzymes, including endo-1,3-β-glucanase (gene_08200), cutinase (gene_04126), and pectin acetylesterases (gene_08821 and gene_09500). Rather than extensive cell wall degradation, the expression pattern suggests localized remodeling of host cell walls to facilitate penetration and haustorial establishment while preserving host cell viability.

In parallel, several genes involved in redox homeostasis and detoxification, including glutathione S-transferases, endoplasmic reticulum oxidoreductin 1, and FAD-dependent oxidoreductases, were strongly induced, indicating active suppression of host-derived reactive oxygen species. Furthermore, induction of polyamine metabolism genes, particularly spermidine synthases (gene_11766 and gene_06392), suggests a metabolic adaptation supporting sustained biotrophic growth.

Collectively, these pathogen-side transcriptional features highlight a PV01-specific virulence program characterized by immune suppression, controlled host cell wall modification, redox balancing, and metabolic adaptation. These findings extend beyond canonical host response signatures and emphasize isolate-specific mechanisms underlying biotrophic infection by *P. viticola* PV01. (**Supplementary File 8**).

### KEGG pathway and GO functional classification of *P. viticola* transcripts

**Figure 13:**
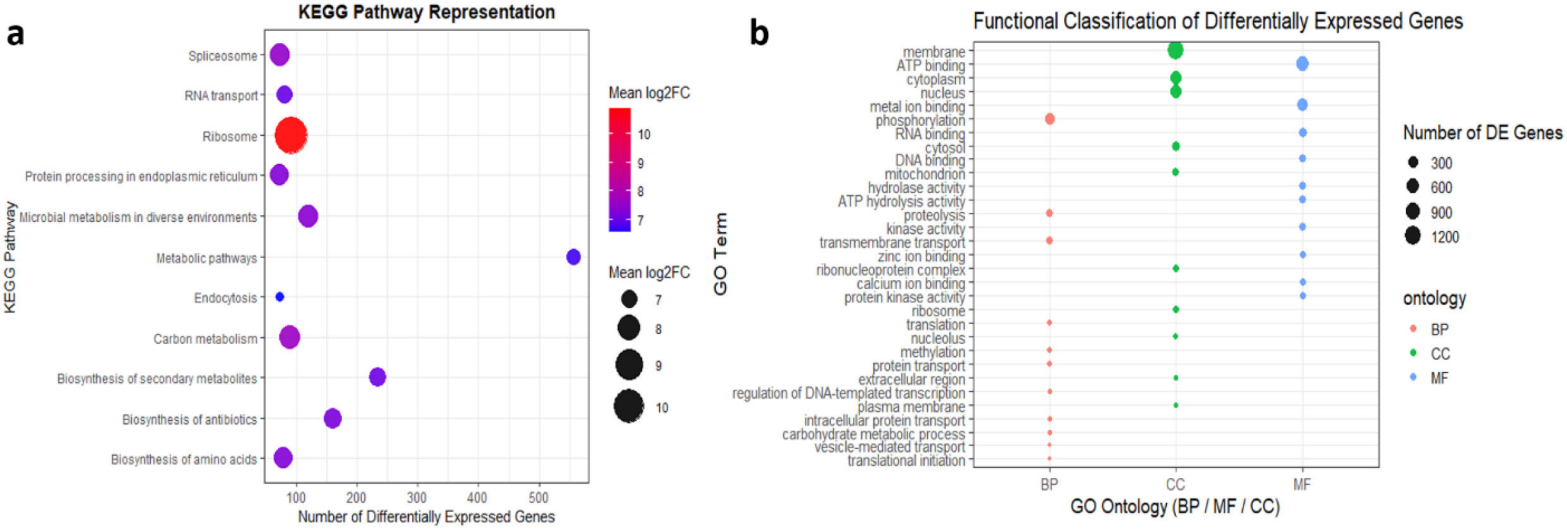
**(a)** KEGG pathway representation of differentially expressed transcripts of *P. viticola*. The x-axis represents the mean log_2_ fold change, while bubble size indicates the number of differentially expressed genes associated with each pathway.**(b)** Gene Ontology (GO) functional classification of differentially expressed genes in *Plasmopara viticola*. Bubble size represents the number of differentially expressed genes associated with each GO term, while colours indicate GO ontology categories: biological process (BP), cellular component (CC), and molecular function (MF).

KEGG pathway representation analysis of differentially expressed genes in *P. viticola* indicated that a large proportion of transcripts were associated with metabolic pathways, including biosynthesis of secondary metabolites, microbial metabolism in diverse environments, and carbon metabolism, reflecting extensive metabolic reprogramming during infection. Pathways related to genetic information processing, such as ribosome, RNA transport, and spliceosome, were also prominently represented and showed high mean log_2_ fold change values, suggesting enhanced transcriptional and translational activity. In addition, pathways involved in protein processing in the endoplasmic reticulum and endocytosis were well represented, indicating active protein maturation, trafficking, and cellular remodeling processes (**Figure 13a**). Complementary Gene Ontology (GO) functional classification further revealed that differentially expressed genes were mainly associated with biological processes related to translation, protein transport, carbohydrate metabolism, vesicle-mediated transport, and regulation of DNA-templated transcription. Cellular component analysis showed predominant localization of these genes to the cytoplasm, nucleus, plasma membrane, ribosome, and mitochondrion, while molecular function annotation highlighted ATP binding, metal ion binding, RNA and DNA binding, kinase activity, and hydrolase activity. Together, these functional annotations illustrate broad metabolic activity and heightened gene expression machinery in *P. viticola* during host infection, consistent with the physiological demands of an obligate biotrophic pathogen (**Figure 13b**).

### qPCR based validation of transcriptome

**Figure 14:**
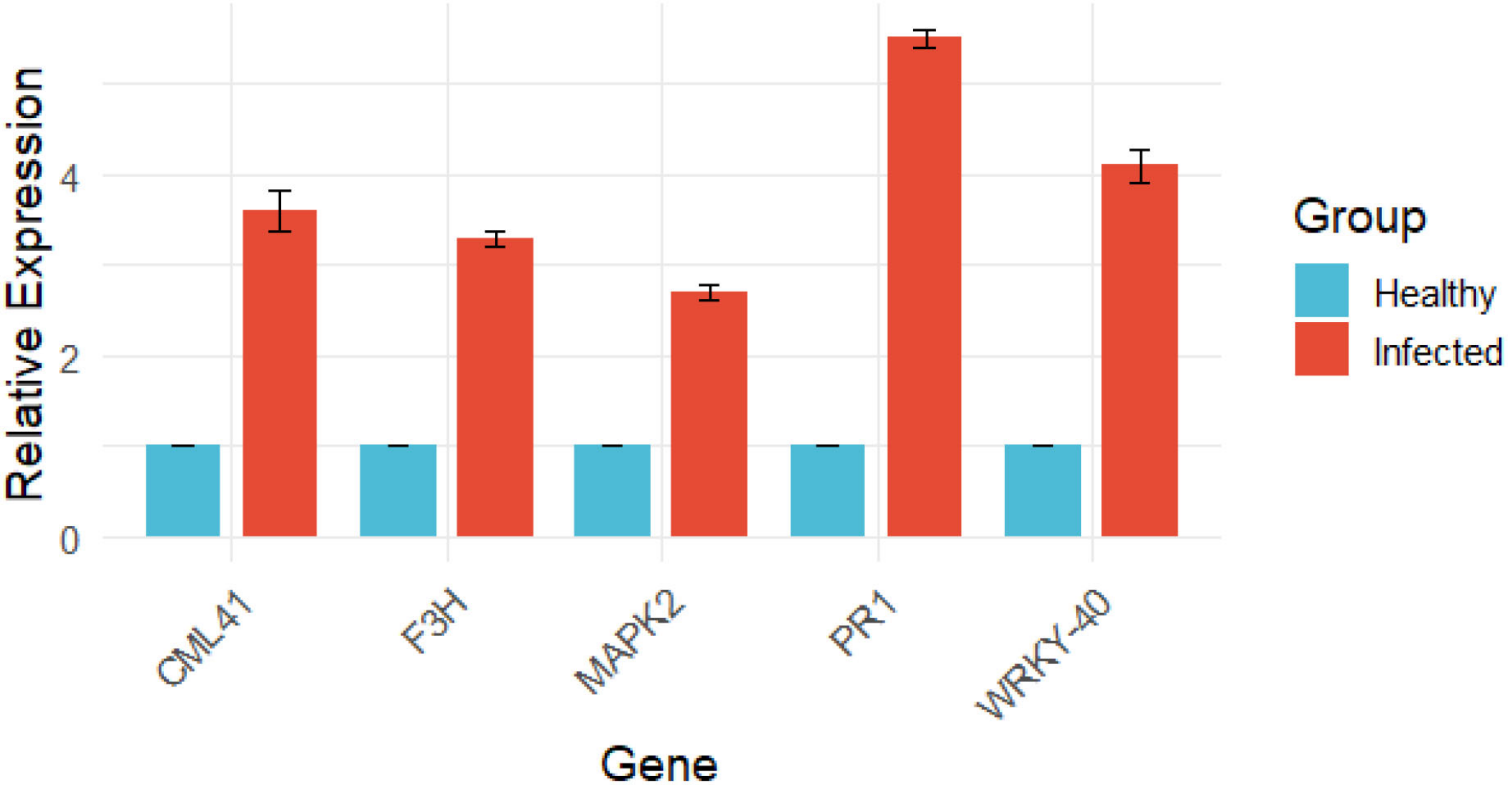
qPCR-based validation of transcriptomic data showing relative expression levels of selected defense-related genes in healthy and downy mildew–infected grape samples. Bars represent mean expression values normalized to the reference gene, with error bars indicating standard deviation, confirming consistency between qPCR and RNA-seq results.

Quantitative real-time PCR analysis was performed to validate the transcriptome data, and the expression trends of all selected genes were consistent with the RNA-Seq results. The qPCR analysis revealed higher transcript levels of PR1, WRKY-40, CML41, F3H, and MAPK2 in *P. viticola*-infected grapevine leaves compared to healthy controls. Among these, PR1 showed the highest upregulation (5.50-fold), followed by WRKY-40 (4.10-fold), CML41 (3.60-fold), F3H (3.30-fold), and MAPK2 (2.70-fold), confirming the reliability of the transcriptome expression patterns (**Figure 14**).

## Discussion

*P. viticola*, the causal agent of grapevine downy mildew, remains a major constraint to viticulture worldwide, particularly under humid conditions. As an obligate biotrophic oomycete, its survival depends on tightly coordinated suppression of host immunity and nutrient acquisition from living tissues. While prior studies have examined individual aspects of *P. viticola-Vitis vinifera* interactions, including resistance mechanisms and effector biology, comprehensive integrative analyses are limited. This study bridges that gap by combining morphological, phylogenetic, genomic, and transcriptomic analyses to provide a detailed view of pathogen virulence strategies and host immune responses.

Morphological examination confirmed characteristic features of infection, including tree-like sporangiophores and biflagellate zoospores (Gessler et al., 2011). Light and scanning electron microscopy revealed extensive branching, monopodial architecture, and hyaline, lemon-shaped sporangia, validating isolate identification. Phylogenetic analyses based on ITS clustered the isolate PV01 with other *P. viticola* isolates, indicating high genetic conservation across populations and limited divergence, likely due to its obligate biotrophic lifestyle (Yin et al., 2017b).

The hybrid genome assembly integrating Illumina and Nanopore reads produced a highly contiguous assembly of 84.1 Mb size with 97% BUSCO completeness is comparable with previous genome reports. Dussert et al. (2016) reported that *P. viticola* isolate INRA-PV221 had 74.74 Mb size with 91% completeness. Similarly Yin et al. (2017a) also stated that hybrid assembly of *P. viticola* isolate JL-7–2 had 101.3 Mb. Despite the genome size differences, all previously reported *P.viticola* genomes had GC content of 44% is consistant with our results. Similarly annotation results are also is consistant with earlier reports of Yin et al. (2017a) and Dussert et al. (2019), where they have reported that 17,014 protein coding genes and 15,960 protein coding genes, whereas in this study we report that *P. viticola* Pv01 genome codes 12,404 protein coding genes.

Functional annotation of the PV01 genome revealed a diverse repertoire of putative virulence-associated genes and secreted proteins, including hydrolytic enzymes, ABC transporters, and candidate effector proteins. This virulence-related gene complement is consistent with patterns reported in earlier *P. viticola* genomic studies. In particular, Yin et al. (2017a) and Dussert et al. (2019) demonstrated that *P. viticola* genomes encode a wide range of pathogenicity-associated proteins, including proteases, glycoside hydrolases, elicitins and elicitin-like proteins, as well as multiple classes of cell wall degrading enzymes. These enzymes notably include pectin esterases, pectin lyases, and phospholipases, which are implicated in host cell wall modification and tissue colonization during infection. The concordance of these functional categories across independent genome assemblies supports the robustness of virulence-related gene predictions in PV01 and underscores the conserved pathogenic toolkit of *P. viticola*.

RxLR and CRN (Crinkler) effectors represent two major classes of cytoplasmic effectors that play central roles in oomycete pathogenicity by modulating host immunity and cellular processes (Morgan and Kamoun, 2007; Stassen and Van den Ackerveken, 2011). In the present study, the PV01 genome encodes a substantial complement of predicted RxLR and CRN effectors, reinforcing their importance in *P. viticola*–grapevine interactions. The RxLR and CRN effector repertoire identified in PV01 is comparable to that reported in earlier *P. viticola* genome assemblies. Previous studies by Yin et al. (2017a) and Dussert et al. (2019) demonstrated that *P. viticola* genomes harbor large and diversified RxLR and CRN effectors. CRN effectors capable of inducing necrosis suggest that *P. viticola* can manipulate host cell death at specific stages to optimize nutrient acquisition while maintaining biotrophy (Birkenbihl et al., 2012).

Transcriptomic profiling of infected grapevine leaves revealed a dynamic host response. Numerous DEGs associated with immune signalling and defense reflected the host’s active struggle against pathogen invasion. Upregulation of pathogenesis-related (PR) proteins, including PR-1, PR-2, PR-4, PR-5, PR-9, and PR-10, underscores their central role in systemic acquired resistance. PR-1 proteins reinforce cell walls and have antimicrobial activity (Breen et al., 2017). PR-2 (β-1,3-glucanases) and PR-4 (chitinases) degrade pathogen cell walls and release elicitors that amplify defense responses (Kumudini et al., 2018); (Taif et al., 2020); (Hou et al., 2022). PR-5 thaumatin-like proteins inhibit spore germination and hyphal growth (Jayasankar et al., 2003), while PR-9 peroxidases catalyze lignification and ROS-mediated pathogen containment (Kaur et al., 2022). PR-10 proteins suppress hyphal growth and spore germination and interact with pathogen receptors (Dadáková et al., 2015).

Secondary metabolism pathways, including flavonoid and stilbene biosynthesis, were activated, with flavanone 3-hydroxylase (F3H) mediating phytoalexin accumulation, consistent with responses observed during *B. cinerea* infection in grapevine and *L. theobromae* in peach (Figueiredo et al., 2015); (Gao et al., 2016). Hormone-mediated signaling via jasmonic acid (JA), ethylene (ET), and salicylic acid (SA) was also induced, coordinating transcriptional reprogramming and reinforcing local and systemic defenses. Calcium signaling, mediated by calmodulins (CaM) and calmodulin-like proteins (CML), integrated defense responses via WRKY and MYB transcription factors to regulate defense genes and lignin deposition (Aldon et al., 2018); (Marchive et al., 2013) (Yuan et al., 2022); (Costa et al., 2023); (Iqbal et al., 2021).

On the pathogen side, *P. viticola* expressed a broad array of effectors and secreted proteins, including RxLR, CRN, NPP1, trypsin-like apoplastic effectors, elicitins, hydrolytic enzymes, and transporters, supporting tissue colonization and immune suppression. Several *P. viticola* RxLR effectors known to express in infected grapvine including PvRxLR159, which suppresses host cell death during early infection, RxLR50253 and PvRxLR28, which are highly induced at early infection stages and promote grapevine susceptibility, and RxLR31154, which enhances infection by targeting the chloroplast protein VpPsbP (Xiang et al., 2016; Lei et al., 2019; Liu et al., 2021; Yin et al., 2022). Similarly, Proteins such as necrosis- and ethylene-inducing peptide 1–like proteins (NLPs/NPP1) and trypsin-like proteases have been reported to play important roles in Plasmopara infection by modulating host cell death and proteolytic processes during pathogenesis (Schumacher et al., 2020; Figueiredo et al., 2023). Transcriptomic evidence indicated early deployment to manipulate host signaling, suppress autophagy (ATG1, ATG5, ATG6, ATG12 and VPS15), and facilitate nutrient acquisition (Sanchez-Garrido and Shenoy, 2021). Coordinated expression of hydrolytic enzymes, vesicle trafficking proteins, and metabolic enzymes highlights *P. viticola*’s sophisticated molecular toolkit for maintaining biotrophy while overcoming host defenses.

In summary, these findings show that *P. viticola* infection triggers strong suppression of photosynthesis and chloroplast-related pathways in grapevine, while simultaneously activating defense responses involving PR proteins, MAPK signaling, phytoalexin biosynthesis, and ROS detoxification. In parallel, the pathogen deploys a wide range of apoplastic and cytoplasmic effectors, hydrolytic enzymes, and metabolic adaptation genes, while suppressing host autophagy, thereby enabling successful colonization and maintenance of biotrophy.

## Conclusion

This study presents the whole-genome assembly of *P. viticola* isolate PV01 from India, providing a comprehensive view of its virulence mechanisms and host adaptation strategies. Through integrated genome, secretome, effector, and dual RNA-seq analyses, we reveal conserved biotrophic orthogroups shared with *Hyaloperonospora arabidopsidis* and lineage-specific genes unique to *P. viticola*, contributing to grapevine specialization. Early transcriptional activation of effector genes, suppression of host autophagy, and reprogramming of chloroplast-associated metabolism were observed, supporting a biotrophic lifestyle. Fifty key virulence and secreted proteins were identified, reflecting the evolutionary dynamics of oomycete gene families. Host transcriptomics indicated early immune recognition, activation of PR proteins, ROS detoxification, hormone-mediated signaling, and structural reinforcement, underscoring a finely balanced molecular interplay between pathogen suppression and grapevine defense.

## Data Availability

The genome sequence of Plasmopara viticola PV01 has been deposited in the NCBI GenBank database under accession number JBUYVZ000000000, BioProject PRJNA1164924, BioSample SAMN43910172.
